# Magnetic‐Guided Delivery of Antisense Oligonucleotides for Targeted Transduction in Multiple Retinal Explant and Organoid Models

**DOI:** 10.1002/advs.202417363

**Published:** 2025-04-25

**Authors:** Xiuhong Ye, Sihui Chen, Wei Xiong, Fan Wang, Hon Fai Chan, Haocheng Lai, Xiangyu Guo, Tingting Yang, Shuhao Shen, Hang Chen, Wenxuan Wang, Guei‐Sheung Liu, Yonglong Guo, Jiansu Chen

**Affiliations:** ^1^ Department of Ophthalmology The First Affiliated Hospital of Jinan University Jinan University Guangzhou 510632 China; ^2^ Institute of Ophthalmology Medical College Jinan University Guangzhou 510632 China; ^3^ Key Laboratory for Regenerative Medicine Ministry of Education Jinan University Guangzhou 510632 China; ^4^ College of Veterinary Medicine South China Agricultural University Guangzhou 510642 China; ^5^ Institute for Tissue Engineering and Regenerative Medicine The Chinese University of Hong Kong Hong Kong 999077 China; ^6^ Division of Life Science Hong Kong University of Science and Technology Hong Kong 999077 China; ^7^ Guangdong Key Laboratory of Non‐human Primate Research Guangdong‐Hongkong‐Macau Institute of CNS Regeneration Jinan University Guangzhou 510000 China; ^8^ Aier Eye Institute Aier Eye Hospital Group Co. Ltd. Changsha 410000 China; ^9^ School of Biomedical Sciences Faculty of Medicine The Chinese University of Hong Kong Hong Kong 999077 China; ^10^ Centre for Eye Research Australia Royal Victorian Eye and Ear Hospital East Melbourne VIC 3002 Australia; ^11^ Ophthalmology Department of Surgery University of Melbourne East Melbourne VIC 3010 Australia; ^12^ Menzies Institute for Medical Research University of Tasmania Hobart TAS 7001 Australia

**Keywords:** ASO, delivery, MNP, organoid, retinal explant

## Abstract

Antisense oligonucleotide (ASO) therapy holds promise in gene therapy but faces challenges due to poor delivery efficiency and limited evaluation models. This investigation employs magnetic nanoparticles (MNPs) to augment the delivery efficiency of ASOs. It assesses their distribution and therapeutic efficacy across various models, including retinal explants from mice and macaques or human retinal and inner ear organoids. Retinal explants from both mice and monkeys are methodically arranged to expose the ganglion cell layer (GCL) or the photoreceptor layer (PL). MNPs markedly enhanced the penetration and targeting of ASOs, resulting in a 60% accumulation in the GCL or 72% in the photoreceptors. Furthermore, an in vitro biomimetic model of the neuroretina‐RPE/choroid‐sclera complex is developed to examine ASO distribution under dynamic flow conditions. Moreover, the utilization of MNP‐assisted ASO‐Cy3 markedly enhanced transfection efficiency within human retinal and inner ear organoids, resulting in an increase in positively transfected cells to 60% and 70%, respectively. Here, for the first time, an MNP‐explant‐organoid platform is carried out for the promotion of ASO transfection efficiency, therapeutic screening and targeted delivery. This development paves the way for investigating novel gene therapy strategies targeting retinal diseases.

## Graphical abstract

Magnetic field‐guided MNP@ATR‐ASO nanocomplexes facilitate the targeted delivery of ASOs to ganglion cells or photoreceptors within retinal explants, thereby enhancing the efficacy of ASOs in correcting pre‐mRNA splicing abnormalities. Furthermore, the nanocomplexes improve ASO penetration and delivery in human retinal and inner ear organoid models, presenting an effective targeted delivery strategy for RNA‐based therapies in retinal diseases. MNPs: magnetic nanoparticles; ASO: Antisense oligonucleotide.

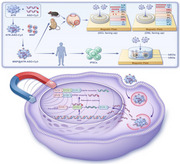

## 1 Introduction

In recent years, antisense oligonucleotide (ASO) therapy has attracted considerable interest due to its potential in addressing ocular diseases, such as inherited retinal diseases, glaucoma, and dry eye disease.^[^ ^1^ ^]^ASOs offer a versatile approach for rectifying splicing abnormalities induced by gene mutations, achieved by targeting specific mRNA sequences.^[^ ^2^ ^]^ This process facilitates restoring normal gene expression through exon skipping or retention.^[^ ^3^ ^]^ Nevertheless, the intricate architecture of retinal cells and the blood‐retina barrier present substantial obstacles to efficiently delivering ASOs to retinal tissues. ^[^ ^4^ ^]^This barrier significantly impedes the direct administration of therapeutic agents.^[^ ^5^ ^]^

Recent advancements in nanotechnology, specifically the utilization of magnetic nanoparticles (MNPs), have demonstrated the potential to address challenges associated with drug delivery.^[^ ^6^ ^]^ Empirical studies have reported considerable progress in employing MNPs for retinal research. For instance, magnetic transfection techniques have been effectively applied to animal retinal explant models, facilitating successful RNA interference.^[^ ^7^ ^]^ Additionally, MNPs have been integrated into eye drop formulations to achieve targeted drug delivery to the retinal photoreceptor layer (PL).^[^ ^8^ ^]^ Their application has been expanded to include the sustained release of drugs and magnetic manipulation within the vitreous of live bovine and rabbit models, thereby illustrating the potential for magnetic regulation of drug distribution. Moreover, MNPs have been utilized to transport various adeno‐associated viruses (AAVs), enhancing the selective transduction of specific retinal layers or photoreceptor cells in porcine explants and whole eyes.^[^ ^9^ ^]^ Consequently, utilizing MNPs in ASO delivery presents significant potential for facilitating the traversal of physiological barriers by ASOs to attain adequate drug concentrations within specific retinal cells impacted by the disease, thereby augmenting therapeutic efficacy.

Furthermore, advancing diverse biomimetic in vitro retinal models is essential, given that the delivery efficiency and therapeutic outcomes of ASOs will likely be affected by the physiological barriers posed by the tightly interconnected retinal cells and the multilayered cellular architecture.^[^ ^10^ ^]^ Traditional ASO drug screening predominantly utilizes minigene systems and animal models.^[^ ^3^, ^11^ ^]^ However, minigene systems frequently function under non‐physiological conditions, resulting in inaccurate expression of splice variants and limited reproducibility of experimental outcomes.^[^ ^12^ ^]^ Conversely, animal models encounter challenges such as interspecies differences and ethical considerations.^[^ ^13^ ^]^ Consequently, there is a critical need to develop in vitro 3D tissue models that more accurately replicate physiological characteristics.^[^ ^14^ ^]^ Retinal explants, which maintain the integrity of multilayered physiological barriers and cell‐cell interactions, mitigate errors related to cell transfection efficiency and variability in drug response, and are amenable to in vitro culture,^[^ ^15^ ^]^ have been widely utilized in retinal disease modelling,^[^ ^16^ ^]^ retinal‐specific cell biology research,^[^ ^17^ ^]^ drug screening and toxicity evaluation,^[^ ^18^ ^]^ as well as assessments of AAV transduction performance.^[^ ^9^, ^19^ ^]^ Furthermore, patient‐derived organoid models effectively mirror the pathogenic mechanisms present in patients and replicate the pathophysiological changes observed during disease progression.^[^ ^20^ ^]^ Consequently, employing organoid models to assess the efficiency of ASO delivery may more accurately simulate the human environment, thereby enhancing the probability of successful clinical translation.^[^ ^21^ ^]^

The primary aim of this study is to formulate a comprehensive and universally applicable delivery strategy employing MNPs to augment the delivery of ASOs to retinal tissues, thereby enhancing therapeutic outcomes in disease models. This will be achieved through the development of innovative retinal in vitro culture models, including retinal explants from mice and macaques that maintain the multilayer microstructure of the retina, as well as human‐derived retinal and inner ear organoid models that accurately mimic the microenvironments and sensitivities of human tissues. These models will be employed to ascertain the optimal experimental parameters for ASOs, perform a series of image analysis at different times in 1 month, and elucidate the dynamic distribution of ASOs across various retinal explant layers. Importantly, after the delivery of ASOs via MNPs, the study will evaluate the activation state of microglia in macaque retinal explants to assess the safety of this method for clinical application, which is a critical factor in translating therapies into patient care. Furthermore, this study aims to assess the delivery efficiency and targeting capability of magnetic nanoparticle MNP‐assisted ASOs, specifically evaluating the splicing regulation efficacy of QR‐421 in cultured retinal tissues.^[^ ^3a^ ^]^ This investigation will offer valuable insights into the feasibility of this combined approach for treating inherited retinal diseases. Integrating MNPs into the ASO delivery system constitutes a substantial advancement in pursuing effective gene therapy for retinal disorders.

## 2 Results and Discussion

### 2.1 Optimization of ASO Delivery Efficiency in Mouse Retinal Explants

To investigate the impact of the retinal physiological barrier on the uptake and delivery of ASOs and to examine the cellular uptake of ASOs in different retinal layers, we employed 2′‐O‐methoxyethyl (MOE) ribose modifications and fully phosphorothioated antisense RNA molecules, which were terminally labelled with Cy3 fluorescence for tracking purposes (ASO‐Cy3). Initially, mouse neuroretinas were cultured in suspension in the medium, followed by the uniform addition of the prepared transfection reagent‐ASO‐Cy3 complex (Figure S1A, Supporting Information). The explant culture method is depicted in the Supporting Information (Figure S1B, Supporting Information).

To further optimize delivery efficiency, we compared the transfection efficiencies of five commercial transfection reagents in retinal explants. Images were captured on days 2 and 7 post‐transfection, and immunostaining was performed on day 7 (Figure S1C, Supporting Information). Advanced DNA RNA Transfection Reagent (ATR) exhibited the highest transfection efficiency, followed by RNA TransMate (RTM), and Lipofectamine RNAiMAX (LRM) ranked third. In contrast, siTIAN 2.0 (SIT) and INTERFERin (INT) demonstrated significantly lower positive transfection efficiency (Figure S1D, Supporting Information). Therefore, ATR was selected as the transfection reagent for subsequent retinal explant experiments (Figure S1E, Supporting Information).

Subsequently, ATR was employed to transfect retinal explants with four distinct concentrations (10, 50, 100, and 200 nM) of ASO‐Cy3 to assess the distribution of ASO across retinal cell layers (Figure S2A, Supporting Information). Statistical analysis of the fluorescence intensity in the 200 nM ASO‐Cy3 group revealed differential permeability of ASO‐Cy3 across all cell layers, with a notable accumulation in the photoreceptor inner and outer segments (IS/OS) (Figure S1F,G, Supporting Information). The fluorescence intensity within the PL exhibited a concentration‐dependent increase, reaching its maximum on days 7 and 14 post‐transfection, followed by a gradual decline by day 28. In contrast, fluorescence within the GCL remained relatively stable, continuing to rise until day 28 (Figure S1H, Supporting Information). Enlarged images demonstrated that a minor quantity of ASO penetrated the inner nuclear layer (INL) and GCL (Figure S1I, Supporting Information). At the same time, the majority was predominantly localized within the inner and outer segments of the PL. This distribution confirmed the colocalization of ASO‐Cy3 with labelled rhodopsin in the outer segments of the PL (Figure S1J, Supporting Information). Considering the dynamic distribution of ASO within the explants, we found that the separation between PL and retinal pigment epithelial (RPE) cells facilitated the direct exposure of PL to the culture medium, enabling active uptake of ASO. As a result, ASO predominantly accumulates within the PL initially before gradually disseminating to the outer nuclear layer (ONL) and progressing to the INL and GCL. The inner limiting membrane (ILM) and nerve fiber layer (NFL) present a barrier effect, impeding the efficient absorption of ASO by the GCL. This observation underscores the role of the ILM and NFL as significant resistance factors in the vitreous injection of ASO. These findings are consistent with previously published studies on the transduction of exosomes or AAVs in porcine retinal explants.^[^ ^9^, ^22^ ^]^

Overall, the application of ATR for the transfection of 200 nM ASOs in mouse retinal explants maintained in suspension culture demonstrated high transfection efficiency. This efficiency exhibited a concentration‐dependent relationship, with maximal fluorescence intensity occurring between days 7 and 14 following transfection. The ASOs were predominantly localized within photoreceptor cells, with minimal distribution observed in the ILM and GCL. Therefore, the suspension culture of mouse retinal explants serves as an optimal in vitro model for simulating homogeneous subretinal ASO delivery, enabling continuous static culture for up to four weeks. This model facilitates the optimization of delivery conditions and yields more reliable and physiologically accurate data compared to cell‐based experiments, as the explants preserve the multilayered cellular architecture of the retina.

### 2.2 Magnetic‐Guided Delivery of ASO for Targeted Transduction in Mouse Retinal Explants

To enhance the targeted delivery of ASO in retinal explants, we acquired MNPs from OZ Biosciences (Marseille, France) to facilitate efficient delivery of ASO through magnetic targeting. The morphology of the ATR‐ASO complex was characterized using TEM, revealing spherical particles with an average diameter of ≈140 nm (Figure S3A, Supporting Information). To characterize the complexation of ASO with ATR and MNP nanoparticles, we assessed the hydrodynamic sizes and surface electrostatic charges of the ATR‐ASO and MNP@ATR‐ASO complexes after 30 min of complexation. As illustrated in Figure S3B (Supporting Information), the mean hydrodynamic sizes of ATR‐ASO and MNP@ATR‐ASO particles, as determined by DLS, are 146.6 and 148.9 nm, respectively. The surface electrostatic charges are 11.03 ± 2.969 and 23.9 ± 2.117 mV, respectively (Figure S3C, Supporting Information). Positively charged MNPs attach to these particles' surfaces through non‐covalent interactions, resulting in a nanoscale complex with a millivolt‐level charge that facilitates cellular uptake. The preparation process is shown in Figure S3D (Supporting Information).

**Figure 1:**
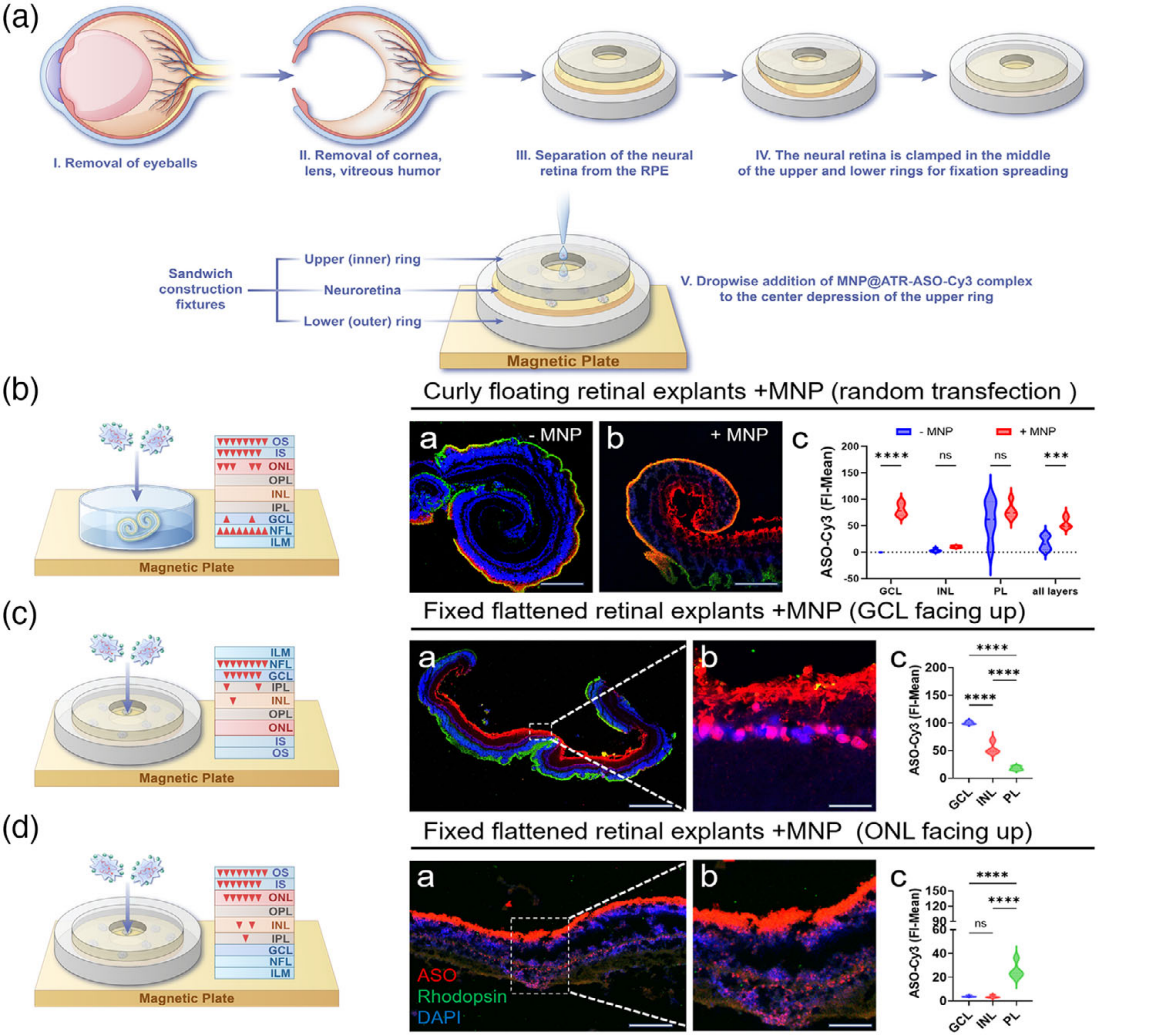
MNP‐Enhanced Delivery and Targeting Efficiency of ASO‐Cy3 in Fixed Flattened Retinal Explants. A) Schematic illustrating the flattening and fixation of mouse neural retinal explants. MNP@ATR‐ASO‐Cy3 nanocomposites are applied to the central fovea of the upper ring for localized targeted transfection. B) Evaluation of the efficacy of transfection with and without the assistance of MNP in floating cultured retinal explants. (B‐a,b) IF image of curled explants. Scale bar: 500 µm. (B‐c) Quantitative analysis of ASO‐Cy3 FI‐mean in different cell layers. C) Efficacy of MNP‐assisted localized targeted transfection with GCL facing up. (C‐a) IF shows ASO‐Cy3 crossing the ILM, primarily accumulating in the GCL. (C‐b) Enlarged images show ASO‐Cy3 distribution in the NFL and GCL. Scale bars: 500 µm, 50 µm. (C‐c) Quantitative analysis of ASO‐Cy3 FI‐mean in different cell layers. D) Efficacy of MNP‐assisted localized targeted transfection with ONL facing up. (D‐a,b) ASO‐Cy3 primarily accumulates in the IS/OS. Scale bars: 500 µm, 100 µm. (D‐c) Quantitative analysis of ASO‐Cy3 FI‐mean in different cell layers. All IF images: ASO‐Cy3 (red), rod cell marker Rhodopsin (green), nuclei stained with DAPI (blue). Bars represent mean ± SD, with n ≥ 3. Statistical analysis was conducted by two‐way ANOVA with Tukey's multiple comparison tests (B); one‐way ANOVA and Tukey's multiple comparison tests (C, D); ns p > 0.05, ***p < 0.001, ****p < 0.0001.

Mouse retinal explants cultured in a floating condition were transfected with 100 nM ASO‐Cy3. After two days, the distribution and average fluorescence intensity of ASO in each retinal cell layer were analyzed, both in the presence and absence of MNP. The findings indicated that MNP significantly enhanced ASO accumulation in the GCL and facilitated a more uniform and dense distribution in the IS/OS (Figure S3E, Supporting Information). Additionally, there was an overall increase in the average fluorescence intensity across the entire retinal explants (**Figure** **1**B). These findings suggest that, under floating conditions, MNP can effectively increase ASO enrichment in the GCL and photoreceptor cells within a shorter timeframe, which is beneficial for assessing the therapeutic efficacy of ASO.

The retinal explants were meticulously flattened and embedded within a collar to optimize the investigation of MNP penetration and targeting capabilities. We administered the MNP‐assisted ASO‐Cy3 complexes (MNP@ATR‐ASO‐Cy3) into the upper chamber of the ring, positioning a magnetic plate beneath to facilitate the guided transition of the complex from the upper to the lower chamber (Figure 1A).

Numerous ocular diseases impact various components of retinal cells, including retinal ganglion cells (RGCs), photoreceptors, and the RPE. In response, we have developed methodologies for the efficient delivery of ASOs into distinct retinal cell types to assess their therapeutic effects. In our experiments, we employed two distinct cellular targeting configurations: one configuration oriented the GCL facing up, specifically designed to penetrate the ILM barrier and target the GCL for ASO delivery (Figure 1C). Conversely, the other configuration positioned the ONL facing up to target the photoreceptor layer (Figure 1D). In cases of retinitis pigmentosa induced by mutations in the USH2A gene, this gene is predominantly expressed at the junction between the inner and outer segments of photoreceptor cells. Mutations in the USH2A gene result in apoptosis and progressive degeneration of photoreceptor cells, leading to a diminished visual field and loss of central vision.^[^ ^23^ ^]^ Consequently, by directing ASO into photoreceptor cells, the concentration of therapeutic agents within these cells can be increased, thereby enhancing the efficacy of ASO‐based treatments.

Figure 1B–D depict the ten retinal layers, delineated by rectangles, while red triangular arrows signify the direction of ASO migration facilitated by MNP traction. The quantity of arrows serves as an approximate indicator of ASO delivery efficiency and the extent of enrichment within specific cellular layers, with a higher number of arrows denoting greater enrichment.

The results indicated that in the GCL facing up group, magnetic force significantly enhanced ASO penetration through the ILM into GCL cells and a significant transfection of ASOs after a 7‐day period (Figure 1C‐a). Immunofluorescence imaging revealed that, although the ILM and NFL absorbed some ASO, a substantial amount successfully crossed the barrier and expressed within GCL cells (Figure 1C‐b). Quantitative analysis of fluorescence intensity indicated that ≈60% of ASO accumulated in the GCL, 28% in the INL, and 12% in the ONL (Figure 1C‐c) (Figure S3F, Supporting Information). In the group with the ONL facing up, a substantial amount of ASOs was observed in the IS/OS region of PL, with a smaller presence in the INL and GCL (Figure 1D). Quantitative analysis of fluorescence intensity revealed that ≈72% of ASOs were localized within the IS/OS, 21% in the INL, and 7% in the GCL (Figure 1D‐c) (Figure S3G, Supporting Information).

In this experiment, fixed and flattened retinal explants were utilized to preserve their viability and enhance the resolution of multilayer retinal cell localization, thereby facilitating precise observation and analysis of nanoparticle penetration. This collar structure demonstrated more convenience compared to conventional models, as it allowed for precise control over the contact area between the nanoparticles and the retina by applying the nanocomplex to the upper chamber, thereby preventing uncontrolled and unstable diffusion.^[^ ^7^, ^9^ ^]^ By flexibly positioning the retinal explant with either the GCL or ONL facing up, magnetic nanoparticles effectively penetrated the NFL and ILM under the influence of magnetic force, targeting the GCL and partially the INL, or increasing ASO enrichment in the PL. These findings corroborate previous research, which similarly demonstrated the efficacy of MNPs in delivering siRNA or AAV to retinal explants.^[^ ^7^, ^9^ ^]^ The ILM, situated between the vitreous body and the retina, constitutes a protein‐rich barrier that significantly impedes the efficient delivery of therapeutic agents from the vitreous cavity to the deeper retinal layers. This study explores the use of MNP in conjunction with magnetic traction to improve the penetration of ASO across the ILM. In vitro retinal models demonstrated that MNPs effectively enhance ASO transduction. This novel methodology holds promise for the treatment of retinal disorders and may offer advantages for future gene and cell therapy applications.

### 2.3 MNP‐Assisted Delivery Enables Safe Targeted Delivery of ASO in Macaque Retinal Explants

The utilization of MNPs markedly improved the targeted delivery of ASOs in murine retinal explants. Considering the structural resemblance of the macaque retina to the human retina, this model presents a superior advantage in terms of biological relevance and potential for clinical translation. Consequently, we expanded the scope of our investigation to assess the efficacy of magnetically‐assisted ASO‐Cy3 delivery in higher primates. During the experiment, the neuroretina was meticulously secured within a collar to maintain a flat configuration, facilitating an accurate evaluation of ASO‐Cy3 delivery. In adherence to the principles of the 3Rs (Replacement, Reduction, Refinement), the neuroretina obtained from a single monkey was subdivided into 12 to 15 segments for fixation.^[^ ^24^ ^]^ This approach significantly minimized the number of animals utilized while ensuring the repeatability of results and reducing individual variability.

**Figure 2:**
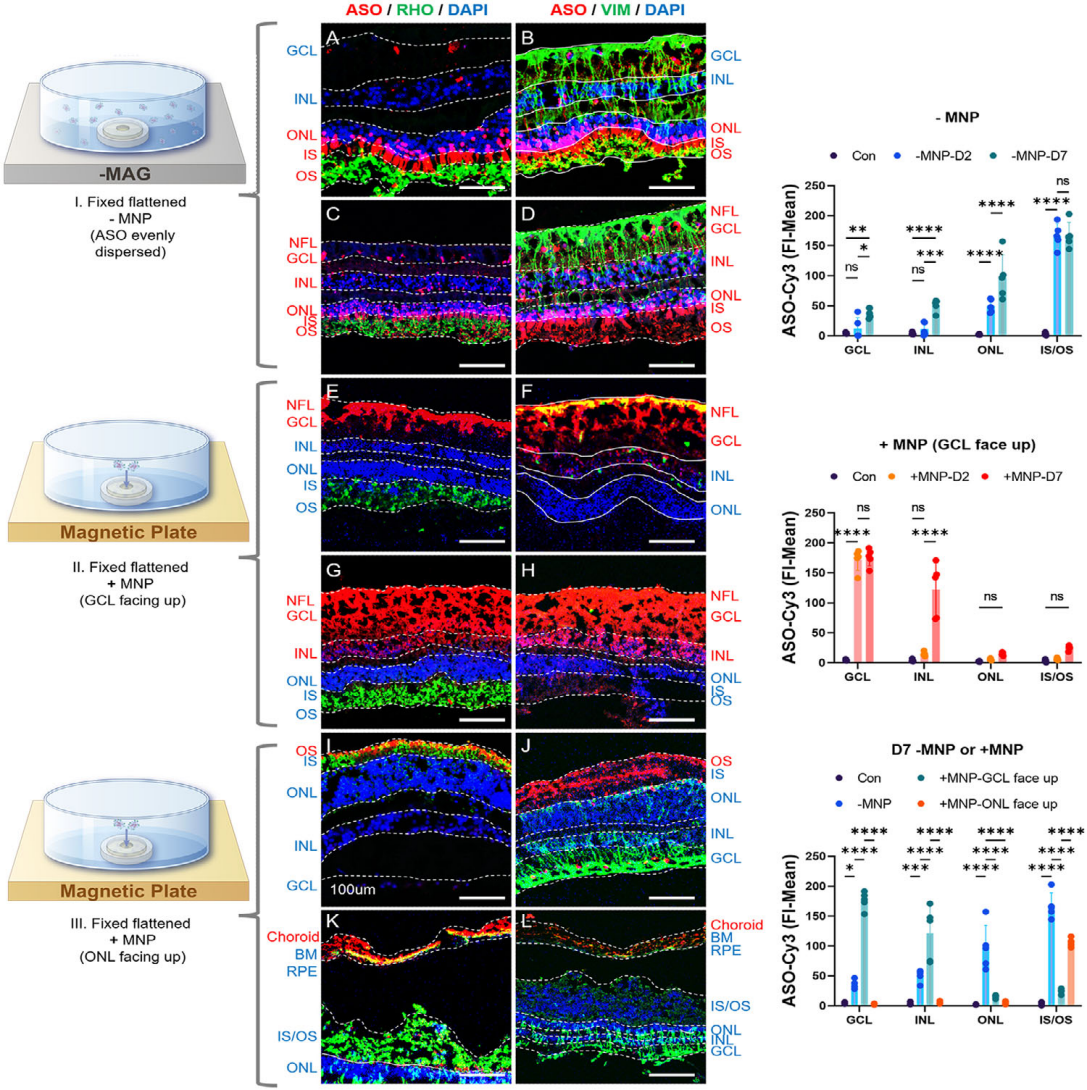
MNP‐Enhanced Delivery and Targeting Efficiency of ASO‐Cy3 in Macaque Retinal Explants. WT macaque neural retinal explants were flattened and fixed. MNP@ATR‐ASO‐Cy3 nanocomposites were applied for localized targeted transfection. A–D) ATR‐ASO‐Cy3 complexes (‐MNP) were evenly distributed in the culture medium of retinal explants. IF images show that ASO‐Cy3 primarily accumulates in the PL and gradually migrates to the ONL, INL, and GCL layers. Statistical analysis reveals the FI‐mean of ASO. E–H) With GCL facing up, MNP facilitated ASO‐Cy3 penetration through the ILM into GCL cells and partially into the INL. I,J) With ONL facing up, MNP concentrated ASO‐Cy3 in the IS/OS. Statistical analysis on day 7 post‐transfection under various conditions. K,L) Choroid/RPE‐retinal explants were fixed with the choroid facing up. MNP@ATR‐ASO‐Cy3 was magnetically guided into the choroid. Scale bar, 100 µm. (A, C, E, G, I, K) Rod cells were labeled with rhodopsin (green); (B, D, F, H, J, L) Vimentin (VIM) staining (green) shows Müller cell fibers spanning the vertical length of the specimen. ASO‐Cy3 (red) and DAPI‐labeled nuclei (blue). (A, B, E, F) are IF images taken on day 2 post‐transfection, while (C, D, G, H, I, J, K, L) are images taken on day 7 post‐transfection. Transfection with non‐fluorescent ASO served as a control group. Statistical analysis was conducted by two‐way ANOVA with Tukey's multiple comparison tests; ns *p* > 0.05, *p < 0.05, **p < 0.01, ***p < 0.001, ****p < 0.0001.

Under non‐magnetic conditions, ASO‐Cy3 primarily accumulated in the ONL and PL 48 h post‐transfection (**Figure** **2**A,B). Furthermore, compared to mouse retinal explants, the ZO‐1 staining in the macaque retina was significantly more pronounced, distinctly delineating the location of the outer limiting membrane (Figure S7A‐c, Supporting Information). After seven days of transfection, immunostaining results indicated that ASO‐Cy3 was concentrated in the IS/OS, with enhanced penetration into the INL and GCL (Figure 2C). Statistical analysis revealed that by the seventh day, the fluorescence intensity of ASO‐Cy3 had increased across all retinal cell layers, with the most significant accumulation observed in the IS/OS, followed by the ONL, INL, and GCL. Immunohistochemical analysis using vimentin staining demonstrated that Müller cells in transfected explants retained a healthy morphology, extending vertically to the INL (Figure 2B,D). GFAP staining confirmed that transfection did not induce significant glial activation or inflammatory response (Figure S7A‐f, Supporting Information).

Under magnetic conditions with the GCL facing up, ASO‐Cy3 effectively crossed the inner limiting membrane, accumulating in the GCL after two days of transfection (Figure 2E). By day seven, the amount of ASO‐Cy3 crossing the inner limiting membrane increased, with significant accumulation in the GCL and INL (Figure 2G). Notably, compared to mouse retinal explants, ASO in the macaque retina exhibited enhanced penetration, traversing the NFL and ILM to reach the GCL and INL. Quantitative analysis showed that by day seven, the fluorescence intensity of ASO‐Cy3 increased across all retinal cell layers, with a significant increase in the INL compared to day two. While MNPs effectively facilitated ASO penetration into deeper retinal layers, this approach led to morphological changes in Müller cells, with synaptophysin staining showing disordered, shortened, and thickened structures (Figure 2F,H).

Under magnetic conditions with the ONL facing up, ASO‐Cy3 remained concentrated in the PL and did not penetrate the ONL even after seven days of transfection (Figure 2I) (Figure S7B‐b, Supporting Information). This observation suggests that the barrier properties of the outer limiting membrane (OLM) effectively confine ASO‐Cy3 to photoreceptor cells, preventing its entry into the ONL. Such selective localization is advantageous for the targeted delivery to photoreceptor cells. Staining of vimentin in explants showed that Müller cells maintained their vertical structure, extending from the GCL to the INL (Figure 2J). A comprehensive statistical analysis on the seventh day of transfection revealed that the group without magnetic nanoparticles (‐MNP) displayed a diffuse distribution across all cell layers. In contrast, in the group with magnetic nanoparticles and the GCL facing up (+MNP‐GCL face up), ASO was primarily concentrated in the GCL and INL, similar to the pattern seen with vitreous injection. In the group with magnetic nanoparticles and the ONL facing up (+MNP‐ONL face up), ASO mainly accumulated in the IS/OS, with minimal presence in other retinal layers. This mimics subretinal injection.

ASO‐Cy3 did not cross Bruch's membrane under magnetic conditions to reach the RPE layer even after seven days of transfection (Figure 2K,L). Magnified images revealed synaptophysin staining marked the RPE cells, while ASO‐Cy3 expression was only detected in choroidal cells. This indicates that ASO‐Cy3 did not penetrate Bruch's membrane to reach the RPE cells (Figure S7B‐d, e, Supporting Information).

A comprehensive analysis of the entire retinal section revealed that in regions containing magnetic nanoparticles, vimentin staining marked the cytoskeleton of Müller cells by day two (Figure S8A‐a, b, Supporting Information) and day seven (Figure S8B‐b, Supporting Information). Notably, the length, density, and structure of Müller cells were significantly diminished. In contrast, regions devoid of these nanoparticles exhibited a well‐preserved vertical structure on both day two (Figure S8A‐c, Supporting Information) and day seven (Figure S8B‐a, c, Supporting Information). These findings indicate that the downward traction force exerted by MNPs, when guided by a magnetic field, adversely affects the morphology and cellular structure of Müller cells. Furthermore, applying magnetic nanoparticles did not significantly increase GFAP expression (Figures S7D‐a, b, and S8C‐b, Supporting Information). However, notable GFAP expression was observed at the sites of retinal folds caused by mechanical compression of the collar. This suggests that while magnetic nanoparticles do not induce GFAP upregulation, external pressure and damage to the retina can trigger this response. Despite some morphological changes in Müller cells, there was no microglia activation, indicating that this method is safe and compatible with primate tissues.

Therefore, we validate the adaptability of this transfection method within macaque retinal explants. Our model of macaque retinal organ culture aligns closely with those previously investigated.^[^ ^25^ ^]^ Additionally, our findings indicate that magnetic nanoparticles substantially improve the penetration and distribution of ASO‐Cy3 across retinal cell layers, facilitating targeted delivery to the GCL, INL, or the IS/OS of photoreceptors, contingent upon the orientation employed. While MNP‐assisted transfection caused some morphological changes in Müller cells, it did not lead to significant GFAP upregulation, thereby confirming the safety and targeted effectiveness of the magnetic nanoparticle‐based transfection system.

### 2.4 Magnetic‐Mediated Transfection of ASO into Dynamically Cultured Murine Biomimetic Retinal Complexes

**Figure 3:**
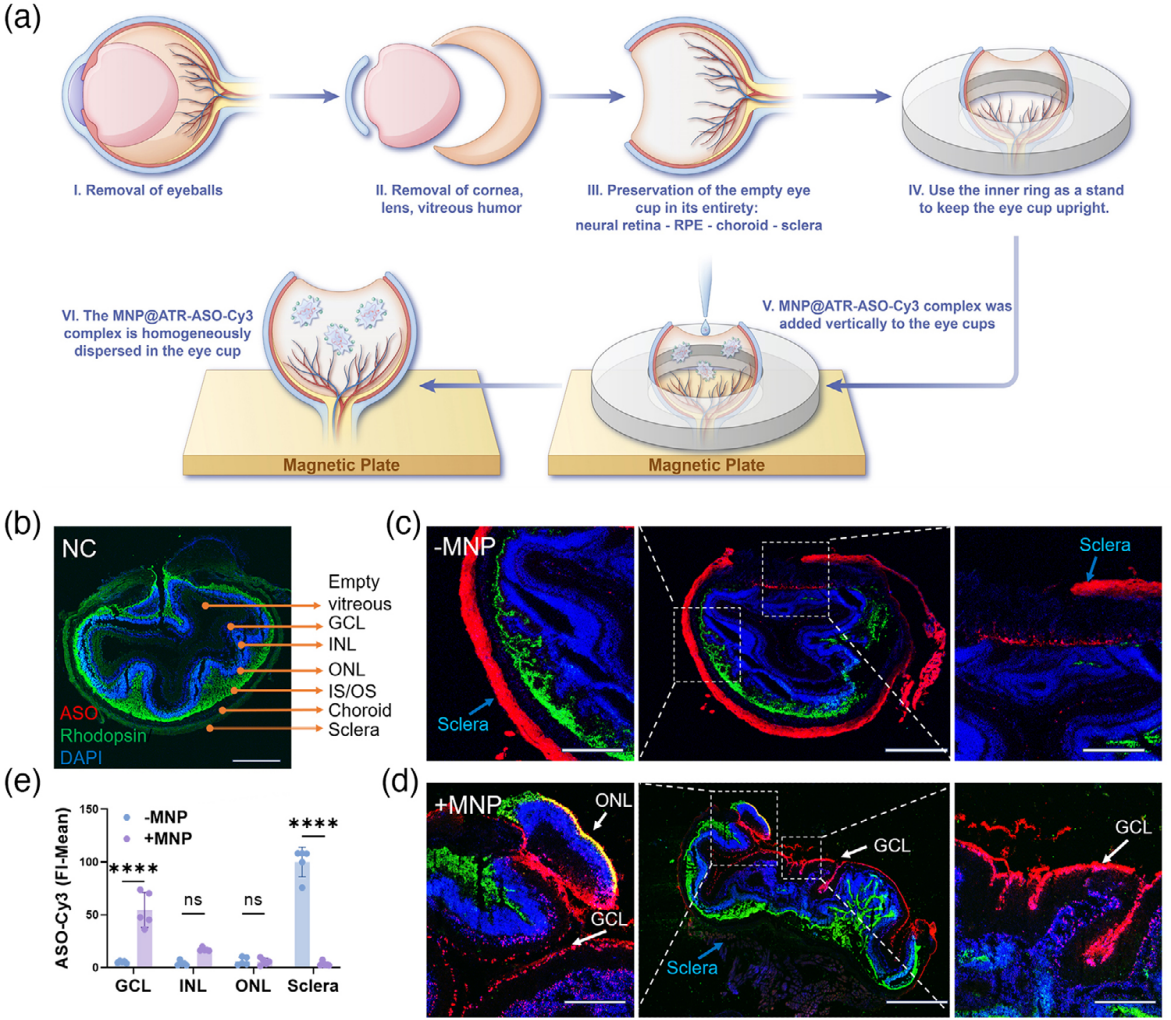
MNP‐Assisted Delivery of ASO in Mouse Retinal‐RPE/Choroid‐Sclera Complex Models. a) Schematic illustration of the preparation, culture, and MNP‐assisted transfection of organotypic mouse retinal models. b) IF images showing the delivery of unlabeled ASO. Scale bar: 500 µm. c) IF images of Cy3‐ASO delivered by ATR (‐MNP). Scale bars: 500 µm (whole retina), 200 µm (magnified view). d) IF images of Cy3‐ASO delivered by the ATR system with MNP (+MNP). Scale bars: 500 µm (whole retina), 100 µm (magnified view). All IF images show ASO‐Cy3 (red), rod cell marker rhodopsin (green), and DAPI‐stained nuclei (blue). e) Quantitative analysis comparing fluorescence distribution of ASO‐Cy3 between ‐MNP and +MNP groups. Bars represent mean ± SD, n = 5. Statistical analysis was conducted by two‐way ANOVA with Tukey's multiple comparison tests (E); ns *p* > 0.05, ****p < 0.0001.

Retinal explants are valuable models for studying how nanoparticles penetrate the retinal barrier, but they lack the biomechanical support of a complete eye. To overcome this limitation, we developed a biomimetic retinal model that maintains the integrity of neural retina‐RPE/ choroid‐scleral tissues. The MNP@ATR‐ASO‐Cy3 complex was introduced into the hollow vitreous cavity, and a magnetic plate was positioned beneath it (**Figure** **3**a).

Our results showed that after removing the vitreous, the neural retina exhibited folding and wrinkling, partially detaching from the RPE (Figure 3b). Without MNPs, the ATR‐ASO‐Cy3 complex primarily accumulated in the sclera and surrounding tissues, with limited penetration into the neural retina (Figure 3c). However, the presence of MNPs significantly improved ASO penetration, allowing effective entry into the GCL and partial penetration into the INL (Figure 3d). Fluorescence analysis indicated that without MNPs, ASO‐Cy3 fluorescence was mainly confined to the scleral region, while with MNPs, fluorescence was concentrated in the GCL (Figure 3e). This study demonstrates that ASO can be effectively targeted to GCL with the assistance of MNP, thereby enhancing the precision of intravitreal drug delivery.

**Figure 4:**
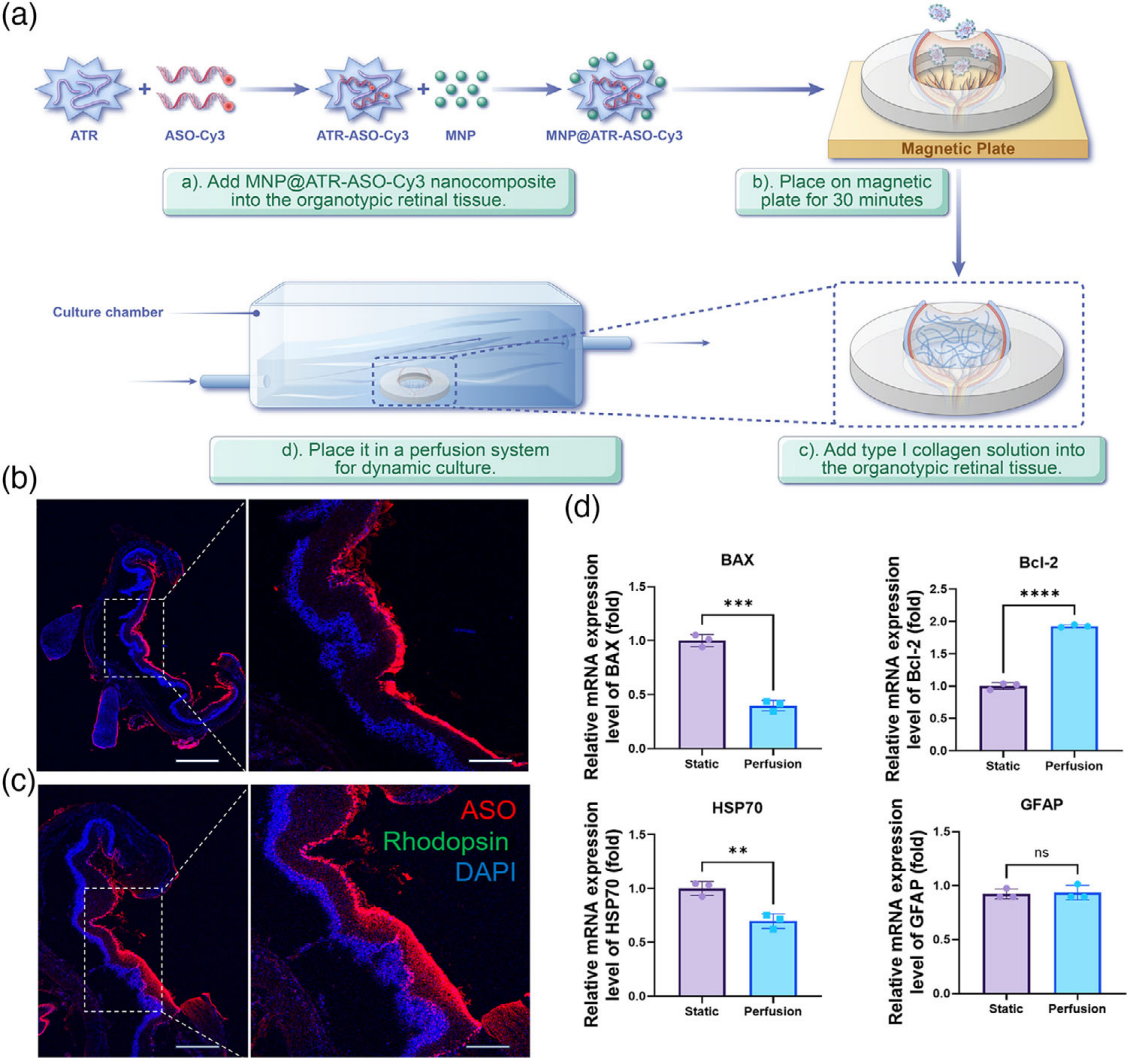
MNP‐Assisted Delivery of ASO in Dynamically Cultured Organotypic Retinal Models. A) Schematic illustration of the process for MNP‐assisted delivery of ASO, including the addition of Type I collagen solution for mechanical support, followed by dynamic culture. B) IF on day 2 shows ASO primarily concentrated in the GCL, with the neural retina closely adhering to the RPE, significantly reducing retinal detachment and folding. Scale bars: 500 µm (whole retina) and 200 µm (magnified view). C) IF on day 7 shows increased migration of ASO from the GCL to the INL. Scale bars: 500 µm (whole retina) and 200 µm (magnified view). All IF images show ASO‐Cy3 (red), rod cell marker Rhodopsin (green), and DAPI‐stained nuclei (blue). D) qPCR statistical analysis reveals significant differences in the expression levels of BAX, BCL‐2, and HSP70 transcripts between static and dynamic perfusion cultures on day 7 (p < 0.05), with no statistical difference in GFAP expression (p > 0.05). Bars represent mean ± SD, with n ≥ 3. Statistical analysis was conducted by unpaired t‐tests (D); ns *p* > 0.05, **p < 0.01, ***p < 0.001, ****p < 0.0001.

The hollow model, lacking vitreous support, displayed retinal detachment and folding. To address this, we filled the vitreous cavity with type I collagen after MNP@ATR‐ASO‐Cy3 transfection to maintain the retina's physiological spherical structure. Type I collagen, the primary component of the vitreous body, is noted for its excellent biocompatibility. It remains in a liquid state at 4 °C and transitions to a solid state within 20 to 30 min at 37 °C, making it an ideal candidate for filling hollow vitreous cavities. The model was then placed in a perfusion system for a dynamic culture (**Figure** **4**A). Immunofluorescence analysis on days 2 and 7 showed significant improvements in retinal flatness and better attachment of the neural retina to the RPE (Figure 4B,C). Quantitative PCR analysis demonstrated that, compared to static conditions, a dynamic culture over a 7‐day period significantly decreased the expression of the pro‐apoptotic marker BAX and increased the expression of the anti‐apoptotic marker BCL‐2. The expression levels of GFAP remained stable, whereas HSP70 levels exhibited a significant reduction (Figure 4D). Our findings indicate that the collagen‐filled retinal complex, when subjected to dynamic culture conditions, significantly mitigates apoptosis and the stress response in retinal cells.

Using the retinal‐RPE/choroid‐sclera complex model filled with type I collagen, we achieved a more biomimetic retinal state that preserved the biomechanical properties of the retina. We demonstrated the effectiveness of MNP‐assisted ASO‐Cy3 delivery through a simulated vitreous injection route, providing an ideal platform for studying drug metabolism and therapeutic effects. Consistent with previous findings, the dynamic culture within a perfusion system enhanced the clearance of metabolic waste and facilitated the delivery of nutrients to deeper retinal tissues, improving retinal vitality and significantly reducing apoptosis in the neural retina.^[^ ^10^, ^26^ ^]^ This extended the evaluation period for ASO metabolism and therapeutic effects. Such an innovative model underscores the potential of MNPs to enhance ASO permeability and overall transfection efficiency in complex 3D tissue environments.

In short, our three retinal explant models each have distinct advantages: the suspension culture model favors transfection parameter optimization, the flat‐fixed model offers a clean and stable 3D environment, and the dynamic culture system maintains a more physiologically relevant biomimetic structure that supports comprehensive studies of metabolism and drug delivery. By integrating these three approaches, we can obtain more comprehensive data to assess the therapeutic effects of nanoparticles and ASO.

### 2.5 The Therapeutic Potential of MNP in Enhancing ASO‐Mediated Splicing Modulation

To evaluate the applicability of the aforementioned mouse retinal in vitro culture model in the study of ASO delivery strategies, we conducted an assessment of its generalizability across different disease models (wild‐type, Ush2a, rd10 mice). Initially, we examined retinal explants from rd10 mice, which serve as a model for rapid retinal degeneration. The findings indicated that the metabolic characteristics and distribution of ASO in these explants were comparable to those observed in wild‐type (WT) mice, with a peak occurring 7 to 14 days following transfection. Due to complete ONL degeneration in rd10 mice, ASO‐Cy3 was mainly located in the INL and GCL regions (Figure S10A,B, Supporting Information). In organotypic retinal complex models derived from rd10 mice, ASO accumulation patterns mirrored those in WT mice, with MNP enhancing ASO concentration in the GCL and INL regions (Figure S10C,D, Supporting Information). Then, we created a mouse model with a pathological intronic point mutation of Ush2a (c.8532‐2A>G), a variant homologous to the human USH2A mutation (c.8559‐2A>G), where retinal explants displayed splicing anomalies akin to those in patients (Figure S9B,C, Supporting Information). Next, we transfected various concentrations of ASO‐Cy3. We tracked fluorescence intensities in different retinal layers over 2 to 28 days, observing similar dynamics between mutant and Wild‐type mice (Figure S11A,B, Supporting Information). These findings demonstrate consistent ASO transfection patterns across different disease models, offering a reliable reference for ASO applications in various conditions.

Furthermore, QR‐421a, an investigational oligonucleotide currently undergoing clinical trials, promotes the skipping of exon 13 in the USH2A gene by modulating splicing, thereby presenting therapeutic potential.^[^ ^3^, ^27^ ^]^ This study investigated the efficacy and therapeutic promise of MNP‐assisted ASO delivery utilizing QR‐421a.

**Figure 5:**
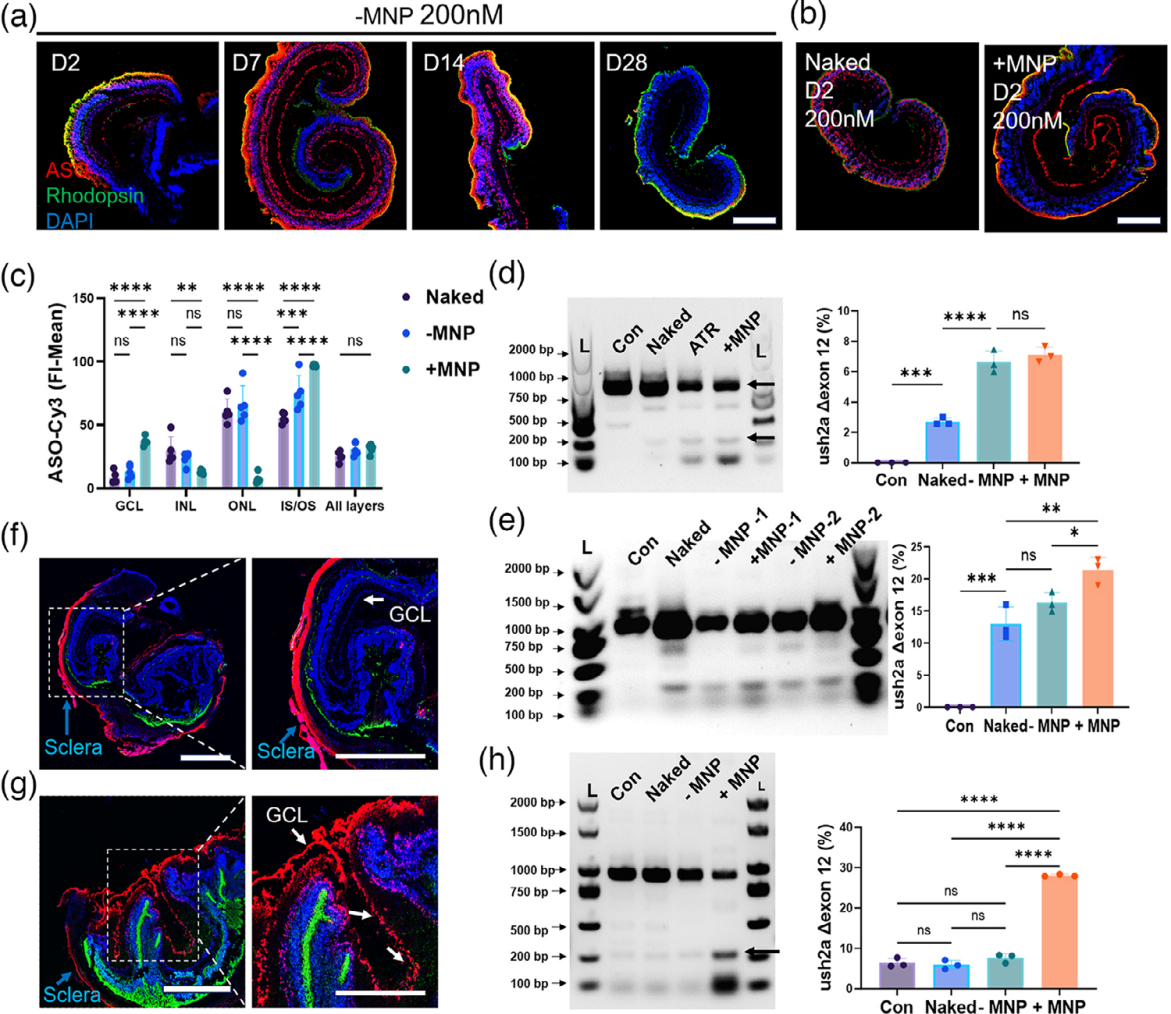
Evaluation of mQR‐421a Splicing Modulation Efficiency in Retinal In Vitro Models. A) ATR (‐MNP) transfection of 200 nM ASO‐Cy3 into retinal explants from Ush2a mice, with IF performed on days 2, 7, 14, and 28 post‐transfection. Scale bar: 500 µm. B) Transfection of 200 nM ASO‐Cy3 into retinal explants using either Naked ATR or MNP‐bound ATR (+MNP), with IF conducted on day 2 post‐transfection. Scale bar: 500 µm. C) Comparison of ASO‐Cy3 FI‐Mean among Naked, ‐MNP, and +MNP groups in retinal explants. D) Transfection of 200 nM mQR‐421a into retinal explants, categorized into Naked, ‐MNP, and +MNP groups. On day 2 post‐transfection, PCR products were analyzed by gel electrophoresis, and exon 12 skipping percentage was calculated. E) Following Cycloheximide treatment, exon 12 skipping percentages were assessed for all three groups. F,G) ASO‐Cy3 transfection into organotypic retinal models, comparing ASO‐Cy3 distribution between ‐MNP and +MNP groups. Scale bars: from left to right: 500, 500, 200, and 200 µm. H) Assessment of mQR‐421a transfection methods in retinal organotypic explants. All IF images show ASO‐Cy3 in red, rod cell marker rhodopsin in green, and nuclei stained with DAPI (blue). Data are presented as mean ± SD, with n ≥ 3. Statistical analysis was conducted by two‐way ANOVA with Tukey's multiple comparison tests (C); one‐way ANOVA and Tukey's multiple comparison tests (D,E,H); ns *p* > 0.05, *p < 0.05, **p < 0.01, ***p < 0.001, ****p < 0.0001.

Following ATR transfection with 200 nM ASO‐Cy3, immunostaining was conducted on days 2, 7, 14, and 28 (**Figure** **5**A). We compared fluorescence distribution and transfection efficiency across groups: naked ASO‐Cy3, ATR transfection without MNP (‐MNP), and MNP‐aided ATR transfection (+MNP) (Figure 5B). The +MNP group showed the highest average fluorescence intensity in the GCL and IS/OS, while lower intensities were observed in the INL and ONL. However, the overall differences in average fluorescence among groups were insignificant (Figure 5C). The findings demonstrated that ASO was enriched in the GCL and IS/OS regions within the +MNP group. However, given that the total transfection amount of ASO remained constant, no significant difference was observed in the average fluorescence intensity between the groups.

In retinal explants derived from mice possessing the homologous mutation of the human USH2A gene, we transfected with 200 nM of mQR‐421a.^[^ ^3a^ ^]^ RT‐PCR analysis revealed exon 12 skipping rates of 3% for the naked ASO group, 6% for the ‐MNP group, and 7% for the +MNP group (Figure 5D). Following RNA degradation inhibition with cycloheximide,^[^ ^3c^ ^]^ all groups showed increased exon skipping rates, with the +MNP group reaching ≈20% (Figure 5E). These results suggest that cycloheximide may enhance the therapeutic efficacy of ASO by inhibiting RNA degradation, underscoring the critical role of sufficient transcript expression in RNA‐based therapies.

In organotypic retinal complex models derived from mice possessing the homologous mutation of the human USH2A gene, the ‐MNP group exhibited significant ASO‐Cy3 accumulation in scleral and scleral epithelial tissues (Figure 5F). In contrast, the +MNP group enriched the GCL and parts of the INL (Figure 5G). Moreover, within the organotypic retinal model transfected with 200 nM mQR‐421a, the +MNP group exhibited a significantly higher frequency of exon 12 skipping compared to the other groups, with an incidence rate of ≈27% (Figure 5H). This finding supports the application of MNP in ASO delivery, particularly in enhancing ILM permeability, laying the groundwork for future clinical applications in retinal diseases.

This study investigates the role of MNPs in enhancing ASO‐mediated splicing modulation therapy. In retinal explants and biomimetic organotypic retinal complex models, MNPs significantly improved short‐term therapeutic outcomes associated with ASO‐mediated splicing modulation. Furthermore, MNPs demonstrated advantages in overcoming physiological barriers, selectively targeting specific cell layers, and promoting splicing modulation.

### 2.6 MNP‐Assisted Delivery of ASO Enhances Transduction Efficiency and Penetration in Human Organoid Models

**Figure 6:**
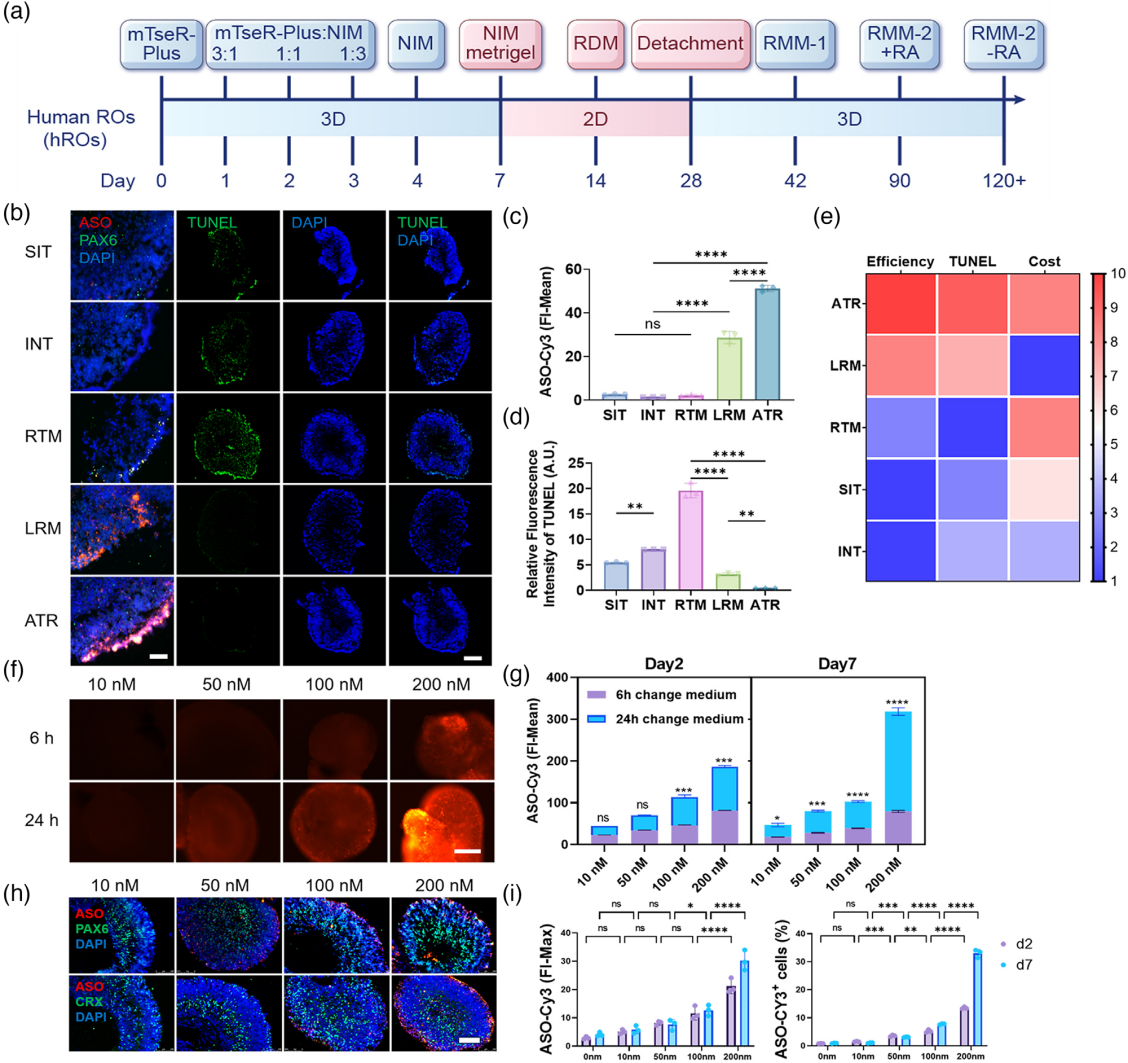
Optimization of ASO‐Cy3 Transfection in hROs. A) Schematic illustrating the differentiation of hiPSCs into hROs. B) IF images of hROs on day 7 post‐ASO‐Cy3 delivery using five different transfection agents. ASO‐Cy3 (red) evaluates transfection efficiency, and TUNEL staining (green) assesses apoptotic cells. Scale bars: 50 µm (left), 500 µm (right). C) Statistical analysis of ASO‐Cy3 FI‐Mean in hROs. D) Statistical analysis of relative fluorescence intensity of TUNEL. E) Heatmap ranking of the five agents based on transfection efficiency, TUNEL staining, and cost. F,G) Comparison of hROs IF images with medium replacement at 6 and 24 h post‐transfection. ASO‐Cy3 (red). Scale bar: 250 µm. H) IF images of hROs on day 7 post‐transfection at varying concentrations. ASO‐Cy3 (red), PAX6 (green), nuclei (blue). Scale bar: 250 µm. I) Flow cytometry (FCM) analysis showing maximum fluorescence intensity (FI‐MAX) and percentage of Cy3‐positive cells on days 2 and 7 post‐transfection. Data are presented as mean ± SD, n ≥ 3. Statistical analysis was conducted by one‐way ANOVA and Tukey's multiple comparison tests (C,D); two‐way ANOVA with Tukey's multiple comparison tests (G,I); ns *p* > 0.05, *p < 0.05, **p < 0.01, ***p < 0.001, ****p < 0.0001.

**Figure 7:**
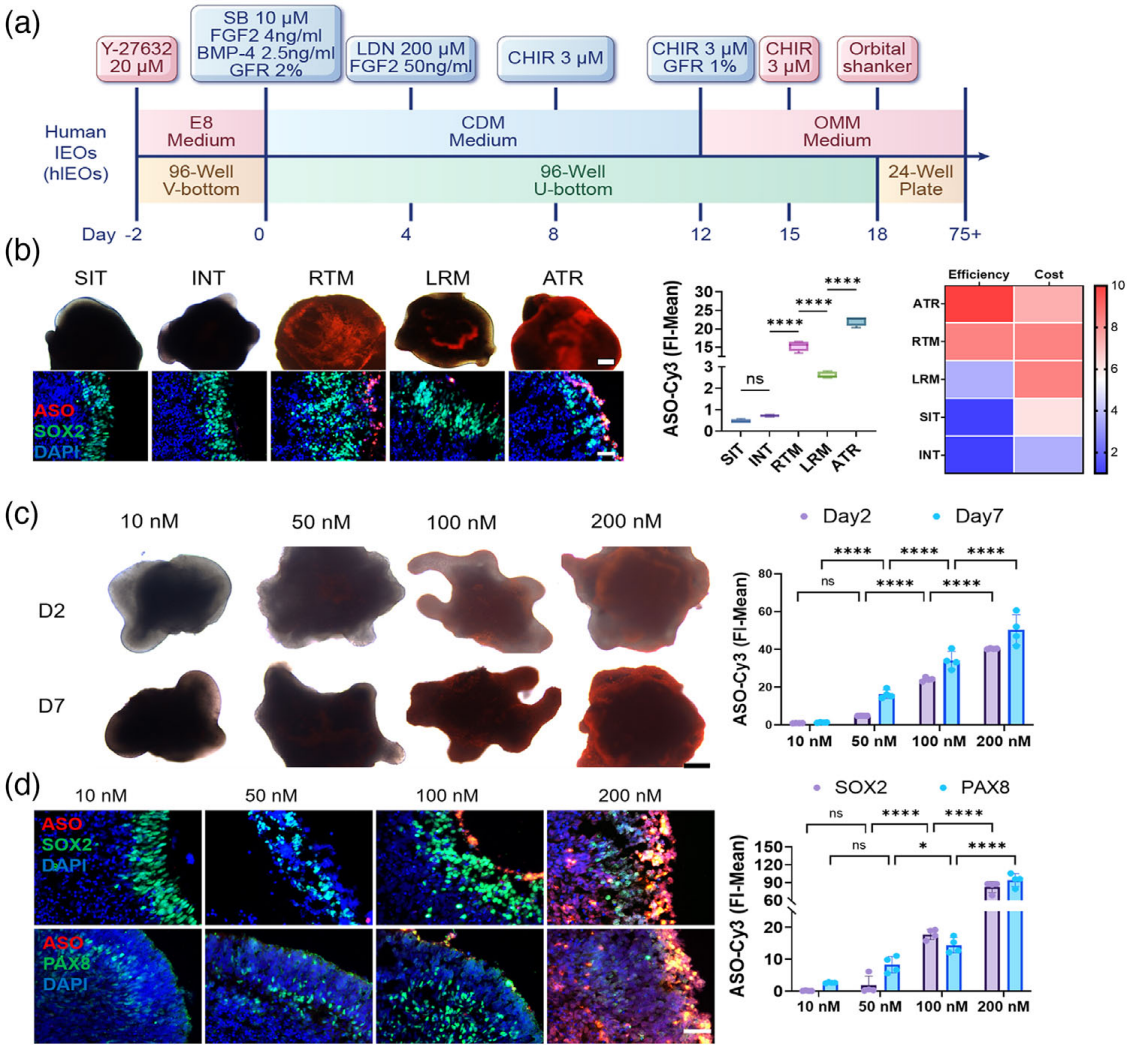
Optimization of ASO Transfection Efficiency in hIEOs. A) Schematic illustrating the differentiation process of hIEOs. B) IF analysis of ASO‐Cy3 (red) FI‐Mean on day 7 post‐transfection using five different agents in hIEOs. Neural progenitor cell marker SOX2 is shown in green. Scale bars: 250 µm (top), 50 µm (bottom). The graph represents the FI‐Mean of ASO‐Cy3 in hIEOs, with transfection agents ranked based on transfection efficiency and cost. C) Transfection of hIEOs with gradient concentrations of ASO‐Cy3 using ATR, with images taken and statistical analysis performed on days 2 and 7 post‐transfection. Scale bar: 250 µm. D) IF of hIEOs on day 7 after transfection with varying concentrations of ASO‐Cy3 using ATR. ASO‐Cy3 is labeled in red, with the neural progenitor cell marker SOX2 (green) and the inner ear progenitor cell marker PAX8 (green). Scale bar: 50 µm. The statistical graph shows the FI‐Mean of ASO‐Cy3 in hIEOs. Data are presented as mean ± SD, n ≥ 3. Statistical analysis was conducted by one‐way ANOVA and Tukey's multiple comparison tests (B); two‐way ANOVA with Tukey's multiple comparison tests (C,D); ns *p* > 0.05, *p < 0.05, ****p < 0.0001.

We developed induced pluripotent stem cells (iPSCs) from patients with the USH2A (c.8559‐2A>G) mutation, which were then differentiated into human retinal organoids (hROs) and human inner ear organoids (hIEOs) to model the disease's pathology and mechanisms (**Figures** **6**A and  **7**A; Figure S9A, Supporting Information).^[^ ^23^, ^28^ ^]^ The hROs are ideal models for studying inherited retinal diseases.^[^ ^20^ ^]^ However, organoids' complex cellular composition and 3D structure present significant challenges for gene transfection. Traditional transfection methods like lipid and electroporation often struggle to achieve safe and efficient gene delivery.^[^ ^29^ ^]^ This study aimed to optimize ASO delivery efficiency with two organoid models associated with Usher syndrome, including hROs and hIEOs.

To enhance the transfection efficiency of ASO‐Cy3 in hROs, we compared five commercially available transfection reagents. Results indicated that ATR had the highest transfection efficiency, followed by LRM and RTM, while SIT and INT were less effective (Figure 6B,C). Further analysis revealed that RTM induced a significantly higher apoptosis rate than the other reagents. SIT and INT showed moderate levels and ATR and LRM resulted in the least cell death (Figure 6B,D). Consequently, based on a comprehensive assessment of transfection efficiency, cytotoxicity, and cost, we also selected ATR for transfection in our organoid models (Figure 6E).

We transfected hROs with varying concentrations (10, 50, 100, and 200 nM) of ASO‐Cy3 and assessed the impact of changing the medium at 6‐ or 24 h post‐transfection (Figure 6F). Statistical analysis showed that changing the medium at 6 h significantly reduced transfection efficiency (Figure 6G). Additionally, observations from day 2 to day 7 indicated that transfection efficiency was dose‐dependent, with a notable increase on day 7 compared to day 2 (Figure S12A,B, Supporting Information). On day 7 post‐transfection, immunostaining revealed ASO localization at the periphery of hROs, with ASO‐Cy3 colocalizing with PAX6 and CRX (Figure 6H). Flow cytometry indicated that in the group transfected with 200 nM ASO, the proportion of Cy3^+^ cells increased from 12% on day 2 to 32% on day 7 (Figure 6I).

Subsequently, we compared the effects of five reagents on hIEOs. Results indicated that ATR exhibited the highest transfection efficiency in hIEOs, consistent with the findings in hROs (Figure 7B). Further analysis found a positive correlation between ATR‐transfected ASO‐Cy3 concentration and transfection efficiency, with day 7 efficiency exceeding that of day 2 (Figure 7C). Immunostaining showed ASO‐Cy3 colocalization with Sox2 and PAX8, particularly in the surface cells of the organoids (Figure 7D).

**Figure 8:**
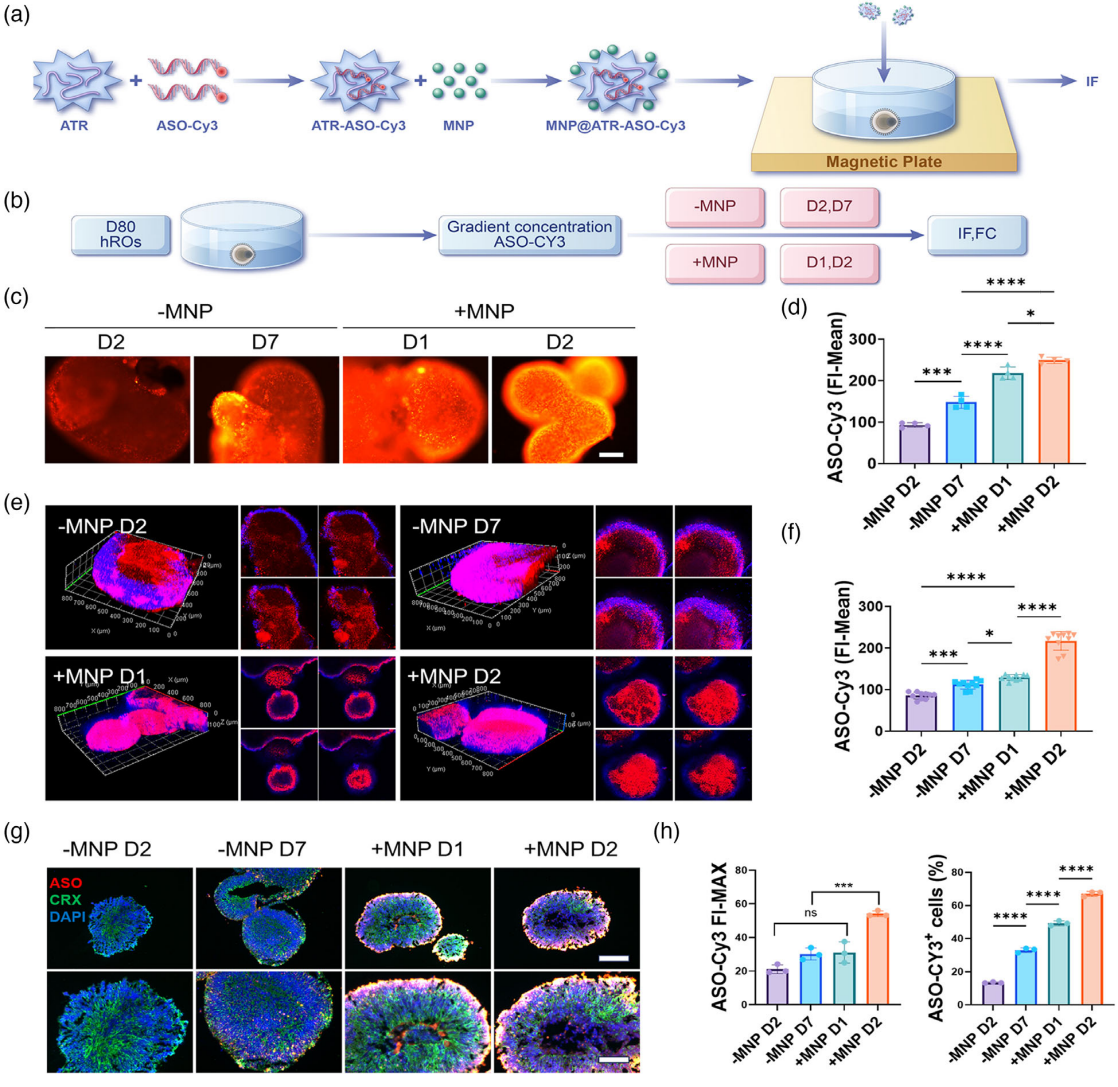
MNP Enhance ASO‐Cy3 Transfection Efficiency and Penetration in hROs. A) Schematic illustrating the preparation process of the MNP@ATR‐ASO‐Cy3 nano‐complex, which is then added to the medium of hROs. B) Schematic illustrating the experimental groups and time points for testing. C,D) IF results show that the FI‐Mean of ASO‐Cy3 significantly increases when combined with MNP. Scale bar: 250 µm. E,F) 3D layer‐by‐layer scanning images of transfected hROs using confocal microscopy, along with statistical analysis of ASO‐Cy3. G) IF results show that MNP significantly enhances ASO‐Cy3 fluorescence intensity in a shorter time, with the photoreceptor precursor cell marker CRX shown in green. H) Flow cytometry results demonstrate that MNP significantly increases ASO‐Cy3 FI‐MAX and the proportion of Cy3‐positive cells in a shorter time. In all IF images, ASO‐Cy3 is labeled in red and nuclei in blue. Data are presented as mean ± SD, n ≥ 3. Statistical analysis was conducted by one‐way ANOVA and Tukey's multiple comparison tests (D,F,H); ns *p* > 0.05, *p < 0.05, ***p < 0.001, ****p < 0.0001.

By optimizing the transfection conditions, we significantly improved the delivery efficiency of ASOs in both hROs and hIEOs. Using ATR to transfect 200 nM ASO yielded satisfactory results by day 7. To further enhance the proportion of positively transfected cells and fluorescence intensity within the organoids, we employed MNPs for assisted delivery in retinal and inner ear organoids. The detailed preparation process for the transfection complex is illustrated in **Figure** **8**A.

In the 80‐day differentiated hROs model, we established four different transfection conditions: 1) no MNP for 2 days (‐MNP D2); 2) no MNP for 7 days (‐MNP D7); 3) MNP‐assisted transfection for 1‐day (+MNP D1); 4) MNP‐assisted transfection for 2 days (+MNP D2) (Figure 8B). Results indicated that the +MNP groups exhibited significantly increased fluorescence intensity on the first‐day post‐transfection, with further enhancement on the second day compared to the ‐MNP groups (Figure 8C,D). Z‐stack confocal microscopy analysis revealed that in the presence of magnetic nanoparticles, ASOs were densely and uniformly distributed on the surface and center of hROs (+MNP group). In contrast, without magnetic nanoparticles (‐MNP group), ASO distribution was less dense and uneven. ASOs struggled to penetrate the dense neural layers but more easily infiltrated the less differentiated zones of RPE cell differentiation. Additionally, the fluorescence intensity in the ‐MNP group was significantly lower than in the +MNP group (Figure 8E,F).

Immunostaining analysis demonstrated that MNP significantly enhanced ASO positivity and permeability in hROs (Figure 8G). Furthermore, flow cytometry analysis indicated that the proportion of ASO‐Cy3 positive cells in the four transfection groups was 18%, 38%, 50%, and 63%, respectively. Notably, the maximum fluorescence intensity in the +MNP groups was significantly greater than in the ‐MNP groups (Figure 8H), suggesting that MNP enhances the penetration of ASO and facilitates nuclear entry, thereby improving overall transfection efficiency.

**Figure 9:**
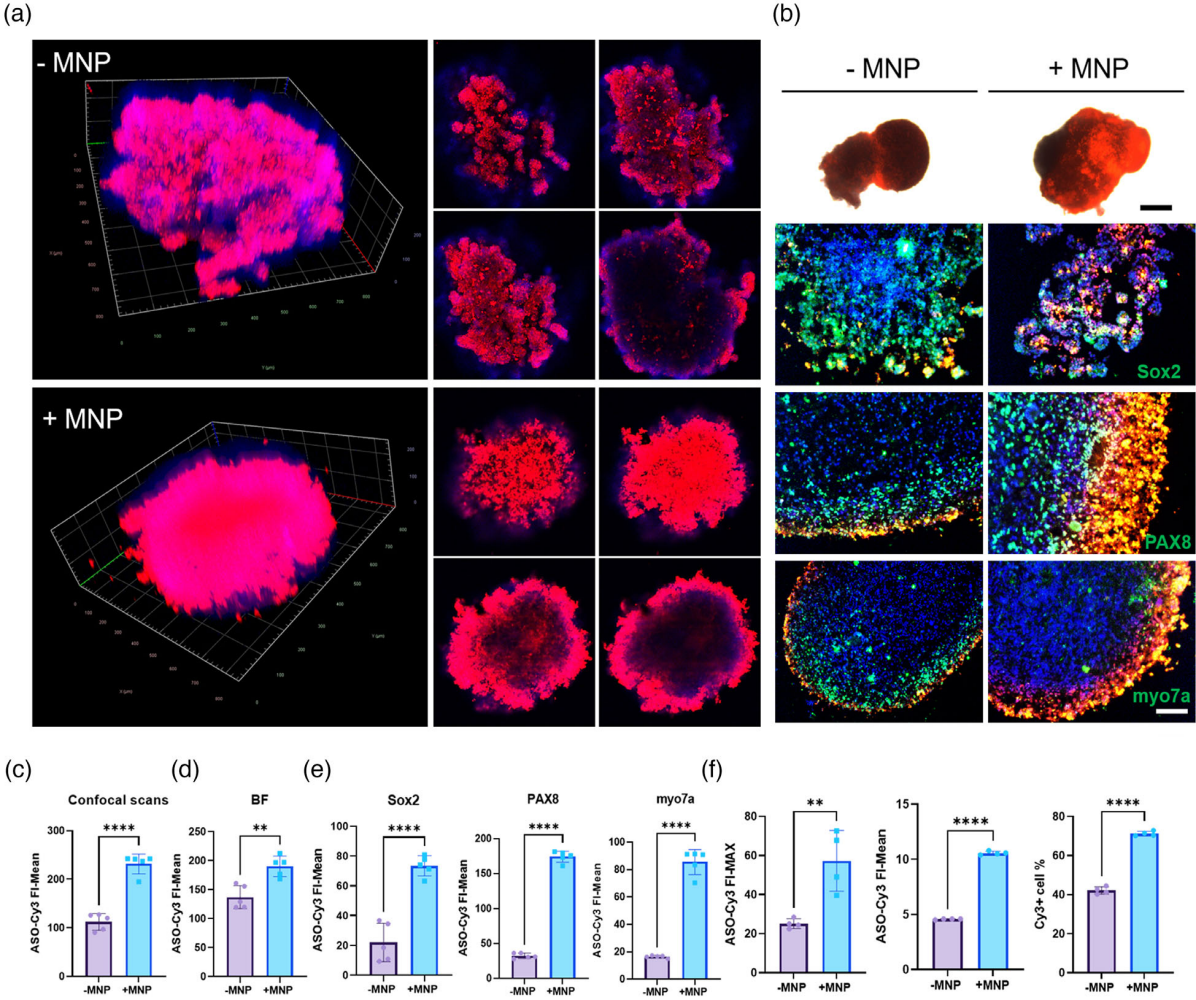
MNP Enhance ASO‐Cy3 Transfection Efficiency and Penetration in hIEOs. On day 7 post‐transfection, confocal scanning, IF, and FCM were performed to evaluate transfection efficiency between ‐MNP and +MNP groups. A) 3D layer‐by‐layer scanning images of hIEOs on day 7 post‐transfection using confocal microscopy. B) Photographs and IF images of hIEOs. Sox2, PAX8, and myo7a are shown in green, while cell nuclei are labeled in blue. Scale bars: 250 µm (top), 100 µm (bottom). C) Slices from confocal imaging were used to calculate the FI‐Mean of ASO‐Cy3, n = 5. D) Analysis of IF imaging for ‐MNP and +MNP groups, n = 5. E) FCM results of ASO‐Cy3's FI‐MAX, FI‐Mean, and the proportion of Cy3‐positive cells, n = 4. In all images, ASO‐Cy3 is labeled in red. Data are presented as mean ± SD. Statistical analysis was conducted by unpaired t‐tests (C‐F); **p < 0.01, ****p < 0.0001.

Next, we performed magnetic‐assisted transfection on hIEOs, evaluating fluorescence intensity and transfection efficiency on day 7 and comparing conditions with and without MNP. Confocal microscopy analysis showed a significant increase in average fluorescence intensity in the MNP‐treated group (**Figure** **9**A,C). Correspondingly, photographs and immunostaining results also showed elevated average fluorescence intensity in the MNP‐treated group (Figure 9B,D,E). Flow cytometry analysis conducted post‐dissociation showed significant increases in both maximum and average fluorescence intensity in the MNP‐treated group. The proportion of Cy3‐positive cells was ≈40% in the ‐MNP group and ≈70% in the +MNP group (Figure 9F), indicating that MNP significantly enhances ASO penetration and transfection efficiency in hIEOs. Furthermore, the positive cell rate in the +MNP group reached 63% and 70% for hROs and hIEOs, respectively. The higher transfection efficiency observed in hIEOs, which may possess a concave surface in contrast to the smooth spherical surface of hROs, underscores the potential of hIEOs as a robust model for evaluating gene therapy.

Overall, organoids' complex cellular composition and 3D structure present challenges for gene transfection. This study is the first to utilize an MNP system in organoid models to enhance ASO permeability and delivery efficiency. We improved ASO transfection efficiency by employing MNPs, immunofluorescence staining, and flow cytometry, achieving 60% ASO‐positive cells in hROs and 70% in hIEOs. This magnetically guided delivery method offers substantial advantages compared to traditional transfection reagents. Additionally, this study is the first to compare the expression differences of USH2A transcripts between hROs and hIEOs at different differentiation time points. qPCR showed elevated USH2A expression in early‐stage hIEOs (Figure S13A, Supporting Information). RT‐PCR results indicated that hIEOs significantly expressed exon 13 by day 30, while hROs required 80 days to reach similar levels (Figure S13B, Supporting Information). This finding enriches the disease model of Usher syndrome. It suggests that hIEOs can express higher levels of USH2A transcripts within a shorter timeframe, providing abundant editable RNA for RNA‐targeted gene therapy, thereby enhancing therapeutic efficacy. The complementarity of these two organoids allows researchers to design multi‐organ experiments to evaluate drug efficacy in the retina and inner ear while examining consistency across different organs, ultimately improving the reliability of experimental results.

## 3 Conclusion

This study thoroughly evaluates the role of MNPs in improving the delivery efficiency of ASOs and suggests enhanced strategies for their delivery. We have made notable advancements in three main areas: First, by embedding retinal explants in collars allowed precise assessment of ASO targeting and penetration. MNPs significantly enhanced ASO delivery to the GCL and PL in both mouse and macaque retinal explants. Second, in complex 3D tissue models, such as hROs and hIEOs, MNPs effectively enhanced the permeability and transfection efficiency of ASOs. Finally, testing the QR‐421a drug in vitro cultured retinal tissue models confirmed MNP technology's potential to boost ASO therapeutic effects, establishing a foundation for clinical applications in retinal diseases.

Retinal explants preserve the intricate multilayered architecture of the retina, rendering them highly suitable for investigating nucleic acid drug delivery mechanisms. Conversely, organoids possess the capability to replicate inherited pathogenic processes while reducing species‐related variability. By integrating these two models, the penetration of ASOs enhanced by nanoparticles can be assessed using mouse explants, followed by preliminary ASO screening. Subsequently, the therapeutic efficacy of ASOs can be evaluated in patient‐derived organoids, facilitating a precise assessment of therapeutic outcomes in human subjects.

Investigating the use of MNPs for ocular diseases in vivo is crucial, but challenges remain. For example, the required 10 µL volume for MNP@ATR‐ASO complexes exceeds the mouse vitreous cavity's capacity. To tackle this issue, vitrectomy in larger animals or the exploration of non‐invasive delivery methods can be employed to assess the suitability of the MNP‐ASO system for in vivo treatment. Designing magnetic plates that match the retina's curvature is essential for even MNP distribution, and adjusting magnetic force based on the distance between the eyeball and skull is necessary for different subjects. These factors will inform our future research.

Future research should explore using MNPs in various in vitro models to improve delivery mechanisms for nucleic acid therapies, particularly in transfecting retinal primary cells and stem cells. By employing diverse retinal culture models and in vitro organoid systems, we can reduce reliance on live animal experiments and improve the precision of preclinical assessments.

## 4 Experimental Section

### Animal

C57BL/6J, rd10 and Ush2a mutation mice were procured from Zhiyuan Biotechnology (Guangzhou, China). All animal experiments were approved by Zhiyuan Biotechnology (Guangzhou, China) (IAEC‐2022010101). Mice aged 8 to 10 weeks were utilized to ensure fully developed eyes for MNP testing. All the mice were placed in an animal house with 12 h of light/darkness and provided with food and water. The eyeballs of the macaque were provided by the Guangdong Key Laboratory of Non‐human Primate Research (IACUC: YXSW‐2023‐009).

### Preparation of ASO Transfection Complexes

ASOs were prepared and stored according to the guidelines provided by Ribobio Biotechnology (Guangzhou, China). These ASOs were diluted to a concentration of 20 µM in RNase‐free water and stored at −80 °C in aliquots. Ribobio also developed a universal negative control ASO, labeled with Cy3 fluorescent dye, which does not target any genes in humans, rats, or mice. The sequences of ASOs used for splicing studies were listed in Table S1 (Supporting Information). Additionally, mQR‐421a was synthesized by GeneScript Biotechnology (Nanjing, China). ATR‐ASO complexes were prepared by mixing ATR with an equal volume of ASO, incubating at room temperature for 15–20 min, and then adding to the culture medium. Preparation of other transfection reagents followed their specific instructions, with details for five reagents in Table S2 (Supporting Information). MNP@ATR‐ASO complexes were prepared following OZ Biosciences' protocol: Magnetic nanoparticles (Catalog # CM21000, CombiMag reagent, OZ Biosciences, Marseille, France) were vortexed and centrifuged. ATR was mixed with ASO and incubated for 15–20 min, then MNP was added. The mixture was either added to the culture medium or applied to retinal explants or organoids. A magnetic plate (Catalog # MF10000, Super Magnetic Plate, OZ Biosciences, Marseille, France) was then used to guide the ASO‐conjugated MNPs toward the magnetic plate. The synthesis scheme of our nanocomposites is based on previously published schemes.^[^ ^7^ ^]^ The collars were made from polytetrafluoroethylene; the dimensions of the collars were illustrated in Figure S3H (Supporting Information).

### Suspension Static Culture of Mouse Retinal Explants

The preparation of retinal explants was performed according to established protocols in the literature.^[^ ^15^, ^30^ ^]^ Mice were humanely euthanized using CO_2_. Each eyeball was then immersed in a PBS solution with 1% antibiotics and antifungal agents at 4 °C. The sclera was stabilized, and a cross incision was made on the cornea. A curved forceps was used to extract the lens through the incision without damaging the eye wall. The cornea was gently lifted with forceps to reveal the retina, which was then delicately extracted with tweezers, discarding any pigmented tissue. The retina was placed in a 12‐well plate for static culture, allowing it to suspend in the medium and naturally curl inward, exposing the photoreceptor outer segments. Figure 1A illustrates this procedure. The retina was cultured in 1 mL of serum‐free neural retinal medium (NRM) as specified in Table S3 (Supporting Information), and maintained at 37 °C with 5% CO_2_ in a humidified environment.

### Comparison of Five Commercial Transfection Reagents

This study evaluated five commercial transfection reagents by preparing them with ASO‐Cy3 according to each product's optimal ratio guidelines. Each reagent transfected ASO‐Cy3 at 100 nM. For example, the ATR protocol involved mixing it with ASO and incubating at room temperature for 15 min. The transfection complex was added to 1 mL of medium with mouse neural retinal explants or organoids and incubated at 37 °C for 2 or 7 days. Fresh medium was replaced 24 h after transfection. Controls included a group with only the transfection reagent and untreated cells. Detection was performed using a confocal laser scanning microscope (Zeiss LSM880).

### Delivery of ASO‐Cy3 in Retinal Explants and Organoids

In this study, naked ASO, ATR‐ASO, or MNP@ATR‐ASO were administered to mouse neural retinal explants or organoids, including WT, Ush2a, and Rd10 variants, in 1 mL of culture medium. Naked ASO or ASO transfection complexes were introduced directly, while the MNP@ATR‐ASO complex was added with a magnetic plate placed under the well plate for 1 h before removal. ASO‐Cy3 was adjusted to final well concentrations of 10, 50, 100, and 200 nM. Explants or organoids were incubated with ASO at 37 °C, with fresh medium replaced 24 h post‐transfection. Retinal explants were cultured for 2, 7, 14, and 28 days, and organoids for 2 and 7 days before immunofluorescence staining. Each condition was tested with a minimum of three samples. A control group used only the transfection reagent without ASO‐Cy3; untreated cells served as another control. Fluorescence images were taken with a fluorescence microscope (Leica TCS SP8), and 3D scans, along with a qualitative assessment of fluorescence distribution, were performed using a confocal laser scanning microscope (Zeiss LSM880).

### Static Culture of Flattened Retinal Explants Post‐Fixation

After removing the eyes, they were stored on ice in a balanced salt solution with 1% penicillin/streptomycin and taken to the lab within 2 h post‐mortem.^[^ ^22^, ^31^ ^]^ All steps were conducted under sterile conditions using a Zeiss Universal S3 dissection microscope. The anterior segment, including the lens, iris, and vitreous body, was carefully cut out at the limbus and cornea and discarded. The monkey retina was divided into 12–15 uniform pieces, each ≈3×3 mm, to match the size of mouse explants. All preparations were from the same retinal region. The neural retina was carefully separated with fine forceps and a microsurgical knife, then flattened and secured in a collar‐shaped holder. The upper collar was designed to fit snugly into the lower collar, with a gap between them large enough to accommodate the neural retina. Figure 1A shows how explants are embedded in collars before being placed in a static 12‐well culture plate, with either the GCL or photoreceptor layer facing up, as needed. Explants were cultured at 37 °C in a 5% CO_2_ humidified atmosphere, with half of the medium replaced every 48 h from day 2 for the static culture group.

### Magnetic‐Guided Delivery of ASO on Flattened Retinal Explants

Retinal explants from mice or trimmed macaque retinas were extracted and flattened, securing the neural retina within a collar‐shaped holder. The GCL or ONL was oriented upward to form a sandwich structure. The tissue was placed in a culture medium without submerging, and MNP@ATR‐ASO‐Cy3 complexes were added to the holder's upper recess. The culture plate was then positioned on a magnetic plate and incubated at 37 °C in a humidified atmosphere of 5% CO_2_ for 60 min to facilitate the downward movement of the complexes through the retina. Then, 1 mL of culture medium was added. After removing the magnet, the medium was changed 24 h later, and the retinal explants were cultured for another 2 or 7 days. The distribution of ASO‐Cy3 in the retina was examined to evaluate the penetration and targeting efficiency of MNP@ATR‐ASO complexes. Control groups included untreated retina, retina treated with only ASO, and retina treated with MNP without ASO. After 24 h, the medium was refreshed, and the retina was incubated for another 48 h. ASO‐Cy3 fluorescence intensity in the transfected explants was qualitatively assessed using a Zeiss LSM880 confocal laser scanning microscope.

### Collection and Dynamic Culture of Neural Retina‐RPE/Choroid‐Sclera Complex Organotypic Models

Mouse eyes were enucleated, and the retina‐RPE/choroid‐sclera complex was isolated by removing the cornea, lens, and vitreous body to form retinal explants. These were positioned upright in a collar to prevent submersion in the culture medium.^[^ ^22^ ^]^ ATR‐ASO or MNP@ATR‐ASO complexes were added to the vitreous cavity, and the culture plate was placed on a magnetic plate. Transfection occurred for 60 min at 37 °C in a 5% CO_2_ environment to ensure effective complex penetration through the retina. After transfection, the magnet was removed, and a 5 mg mL^−1^ type I collagen solution was added to the hollow vitreous cavity and incubated at 37 °C for 20 min to solidify. Culture medium was then added for either static or dynamic culture. The medium was changed after 24 h, with static culture lasting 48 h or 7 days and dynamic culture lasting 7 days. Mouse retinal tissues were cultured for 7 days in a microfluidic system with a 20 µL min^−1^ flow rate using a peristaltic pump, maintained at 37 °C in a humidified 5% CO_2_ environment. The neural retina was then collected to study ASO‐Cy3 distribution, examining the permeability and targeting efficiency of MNP@ATR‐ASO complexes. Control groups were static.

### Differentiation of hROs

The differentiation of hROs follows standardized methods established in existing literature ^[^ ^32^ ^]^ with detailed steps in Table S4 (Supporting Information). The hiPSCs from Nuwacell Biotechnologies, China, were cultured and passaged on Matrigel‐coated plates using mTeSR1 medium (BD Biosciences). On differentiation, day 0 (D0), hiPSCs were enzymatically dissociated into small clusters and suspended in a mTeSR1 medium with 10 µM Blebbistatin (Sigma) to promote aggregation. These aggregates were then gradually shifted to a neural induction medium (NIM), as detailed in Table S5 (Supporting Information).

### Differentiation of hIEOs

The differentiation protocol for inner ear organoids was performed according to established literature ^[^ ^33^ ^]^ with specific culture procedures detailed in Table S6 (Supporting Information). CDM and OMM media compositions are in Tables S7 and S8 (Supporting Information). The hiPSCs were aggregated in a 96‐well V‐shaped plate using CDM medium, then moved to a 96‐well U‐shaped plate on day 4 for continued differentiation. Beginning on day 12, the medium was changed to OMM, and small molecule inducers were added as needed for inner ear organoid differentiation. From day 20 onward, only OMM medium was used, with plates agitated on a shaker. Experiments were conducted after 30 days of differentiation. Meanwhile, organoids at different differentiation stages were collected for RT‐qPCR to measure USH2A transcript levels.

### Real‐Time Quantitative PCR (RT‐qPCR)

RT‐qPCR was used to evaluate gene transcription levels in retinal explants or organoids. Total RNA was extracted from one explant or five organoids per group using the RNAprep Pure Cell Kit (Tiangen), and then converted to cDNA with the ReverTra Ace qPCR RT Kit (Toyobo). RT‐qPCR was conducted with SYBR Green Master Mix (Toyobo) on a LightCycler 480 (Biorad), normalizing gene expression to GAPDH. Changes were calculated using the 2−ΔΔCt method, with statistical analysis from three independent experiments. Primer sequences were in Table S9 (Supporting Information).

### Evaluation of mQR‐421a Modulation Efficiency

The experiment used retinal explants and organotypic retinal‐RPE/choroid‐sclera tissues from USH2A mice. mQR‐421a, mQR‐421a/ATR complexes, or MNP@ATR‐QR‐421a complexes were applied to the retinal cultures or added into the vitreous cavity. The medium was changed 24 h after transfection, and the neural retina was collected 48 h later. RNA was extracted from each retinal explant using the RNAprep Pure Cell Kit (Tiangen) and converted to cDNA with the ReverTra Ace qPCR RT Kit (Toyobo). PCR was conducted on USH2A exons 11 and 13 using the 2 × Phanta Flash Master Mix (Vazyme Biotech, Nanjing, China) to study ASO‐induced alternative splicing. Primer sequences were listed in Table S10 (Supporting Information). Amplified transcripts were visualized on a 1% agarose gel using 0.5× TBE buffer. Exon 12 skipping frequency in the mouse model was quantitatively assessed using a control ASO with similar properties to mQR‐421a but not complementary to Ush2a. Primers and analytical methods were sourced from prior research.^[^ ^3a^ ^]^

### Immunofluorescence Observation

Organoids and retinal explants were carefully harvested after euthanasia and promptly frozen by liquid nitrogen in optimal cutting temperature (OCT) compound for the frozen section. The tissues were frozen‐sectioned (10 µm) and fixed in 4% paraformaldehyde for 15 min. Then, they were rinsed by PBS three times. The nonspecific binding site of section was blocked with ready‐to‐use goat serum (Boster) at room temperature for 1 h before adding primary antibodies at 4 °C overnight. All the antibodies were in Table S11 (Supporting Information). After being washed with PBST (PBS with 0.05% Tween) three times, goat anti‐rabbit Alexa Fluor 488 (1:500) or goat anti‐mouse Alexa Fluor 488 (1:500) were added into the sections for 2 h incubation at room temperature in the dark box, and the fluorescence signal of tissues was recorded by confocal fluorescence microscopy (Zeiss LSM880) and DeltaVision Elite high‐resolution microscope imaging system (GE Healthcare, USA).

### TUNEL

Retinal or organoid samples were frozen, sectioned, and analyzed for apoptosis. The TUNEL assay kit was purchased from Beyotime (Shanghai, China). Sections were fixed in 4% paraformaldehyde for 30 min, permeabilized with 1% Triton X‐100 for 15 min, and incubated in TUNEL solution for 1 h at 37 °C in the dark. The sections were stained with DAPI for 5 min, washed thrice with PBS, and mounted with a fluorescent medium. Images were captured using a Leica fluorescence microscope, and ImageJ software was used to quantify TUNEL‐positive areas, presenting data as relative TUNEL fluorescence intensity.

### Flow Cytometry Analysis

Flow cytometry analyzed retinal organoids transfected with ASO‐Cy3 on days 1, 2, and 7 post‐transfections. It was compared ATR‐ASO‐Cy3 and MNP@ATR‐ASO‐Cy3 complexes for transfection efficiency, focusing on cell positivity rate and peak fluorescence intensity. Organoids were dissociated with papain at 37 °C for 15–20 min, and the single‐cell suspension was analyzed using a BD Accuri C6 flow cytometer (BD Pharmingen). Experiments were conducted in triplicate for reliable results, with additional parameters recorded for thorough analysis using BD Accuri C6 software.

### Statistical Analysis

Statistical analyses were performed using Prism 8 software (GraphPad Software, Inc., La Jolla, CA, USA). Non‐paired, non‐parametric two‐tailed Mann‐Whitney U tests were employed to evaluate p‐values and statistical significance, with p‐values less than 0.05 considered statistically significant. Fluorescence intensity, area calculations, and grayscale analyses were conducted using ImageJ software (National Institutes of Health, Bethesda, MD, USA). All relevant data were available from the authors.

## Conflict of Interest

The authors declare no conflict of interest.

## Author Contributions

G.S.L., Y.G., and J.C. contributed equally as senior authors. X.Y., S.C., and W.X. contributed to the methodology of the work. X.Y. and S.C. managed the softwares. X.Y., W.X., F.W., H.L., and G.L. validated the work. X.Y., T.Y., S.S., H.C., and W.X. performed the formal analysis. X.Y., S.C., W.X., F.W., and S.S. conducted the investigation. H.C., X.G., T.Y., S.S., H.C., and W.X. provided the resources for the work. X.Y., S.C., W.X., F.W., H.L., and Y.G. conducted the data curation for the work. X.Y. and Y.G. wrote the original draft. G.S.L., J.C., S.C., W.X., and H.L. reviewed and edited the manuscript; X.Y. and Y.G. created the data visualization. G.S.L., Y.G., and J.C. supervised the work. X.Y., H.C., G.L., G.S.L., Y.G., and J.C. managed the project, and Y.G. and J.C. secured the funding for the work.

## Supporting information



Supporting Information

## Data Availability

The data that support the findings of this study are available from the corresponding author upon reasonable request.
