# Supplementary material for: Magnetic‐Guided Delivery of Antisense Oligonucleotides for Targeted Transduction in Multiple Retinal Explant and Organoid Models
Source: Adv Sci (Weinh). 2025 Apr 25;12(22):2417363. doi: 10.1002/advs.202417363 (PMC12165039; doi:10.1002/advs.202417363)
Supplement: Supplementary file 1 — Supporting Information [file ADVS-12-2417363-s001.docx]

Supporting Information

Magnetic-Guided Delivery of Antisense Oligonucleotides for Targeted Transduction in Multiple Retinal Explant and Organoid Models

*Xiuhong Ye^1,2^, Sihui Chen^1,2^, Wei Xiong^3^, Fan Wang^5^,* *Hon Fai Chan^10,11^, Haocheng Lai^9^, Xiangyu Guo^8^, Tingting Yang^1,2^, Shuhao Shen^1,2^, Hang Chen^1,2^, Wenxuan Wang^1,2^, Guei-Sheung Liu^4,6,7,12*^, Yonglong Guo^5*^, Jiansu Chen^1,2,3,4*^*

^1^Department of Ophthalmology, The First Affiliated Hospital of Jinan University, Jinan University, Guangzhou, China

^2^Institute of Ophthalmology, Medical College, Jinan University, Guangzhou, China

^3^Key Laboratory for Regenerative Medicine, Ministry of Education, Jinan University, Guangzhou, China

^4^Aier Eye Institute, Changsha, Hunan, China

^5^College of Veterinary Medicine, South China Agricultural University, Guangzhou, China

^6^Centre for Eye Research Australia, Royal Victorian Eye and Ear Hospital, East Melbourne, VIC, Australia

^7^Ophthalmology, Department of Surgery, University of Melbourne, East Melbourne, VIC, Australia

^8^Guangdong Key Laboratory of Non-human Primate Research, Guangdong-Hongkong-Macau Institute

of CNS Regeneration, Jinan University, Guangzhou, China

^9^Division of Life Science, Hong Kong University of Science and Technology, Hong Kong, China

^10^Institute for Tissue Engineering and Regenerative Medicine, The Chinese University of Hong Kong, Hong Kong, China

^11^School of Biomedical Sciences, Faculty of Medicine, The Chinese University of Hong Kong, Hong Kong, China

^12^Menzies Institute for Medical Research, University of Tasmania, Hobart, TAS, Australia

Email: guei-sheung.liu@unimelb.edu.au; guoyonglong@163.com; chenjiansu2000@163.com


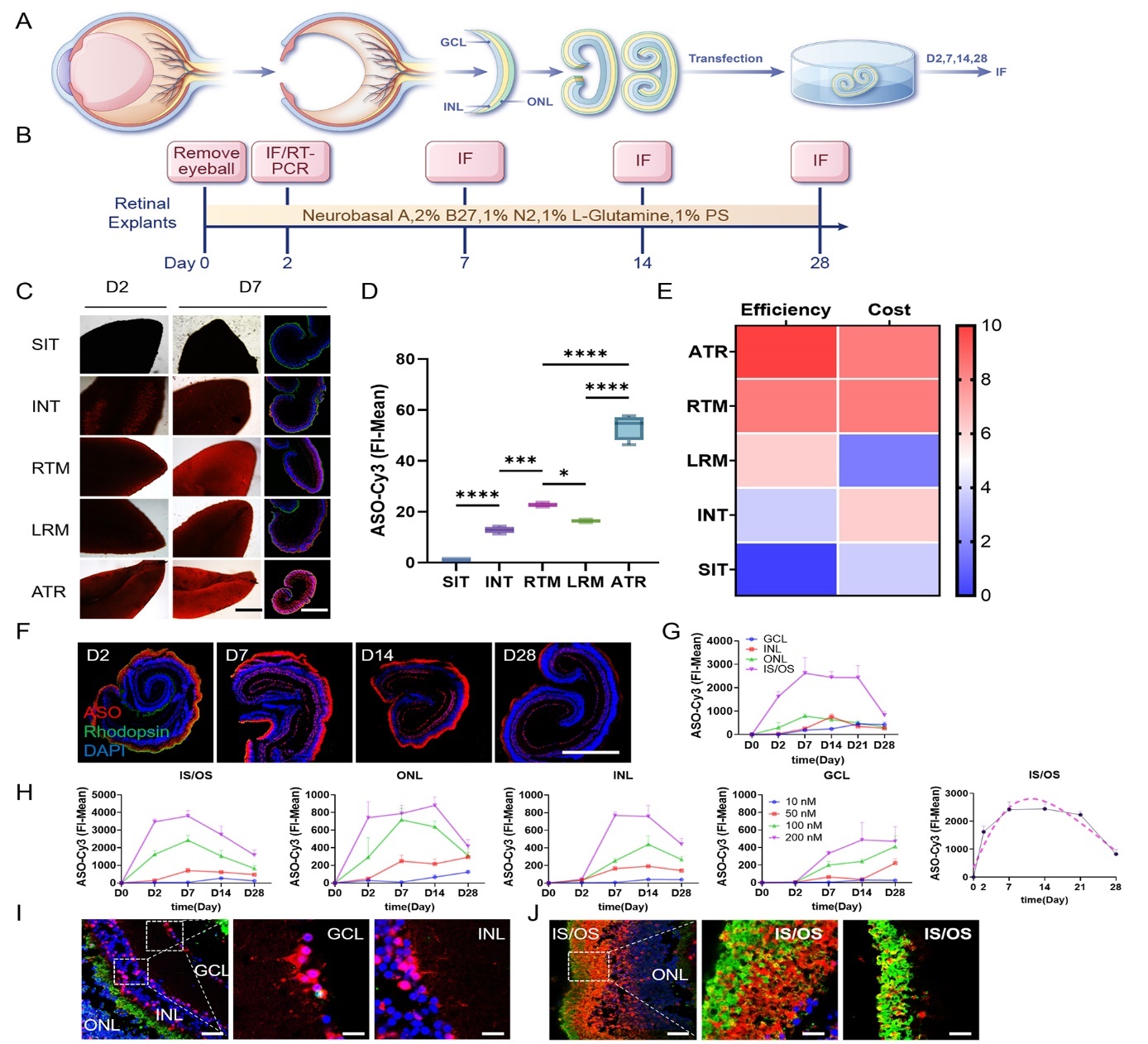


**Figure S1.** Optimization of ASO-Cy3 Transfection Parameters in WT Mouse Retinal Explants. (A) Schematic diagram illustrating the extraction of neural retinal explants, which naturally curl inward, either partially or fully. (B) Methods for culturing retinal explants and analysis time points. (C-E) Transfection efficiency of five commercial reagents was evaluated. (C) Images taken on days 2 and 7, with immunofluorescence staining (IF) performed on day 7. Scale bars: 250 μm, 500 μm. (D) Statistical analysis of average fluorescence intensity (FI-Mean) of ASO-Cy3. (E) Comprehensive ranking of the five transfection reagents, with higher efficiency and lower cost yielding higher scores. (F-H) Transfection of 200 nM ASO-Cy3 using ATR reagent, with IF analysis on days 2, 7, 14, and 28. (F) Scale bar: 500 μm. (G,H) Statistical analysis of FI-Mean. (H) Curve fitting of FI-Mean for the IS/OS layer at 200 nM transfection across different time points. (I, J) Images at day 7 post-transfection at 200 nM. (I) Distribution of ASO-Cy3 in the GCL and INL layers (white box), with scale bars: 100 μm, 20 μm, and 20 μm. (J) Localization of ASO-Cy3 in the IS/OS layer (white box), with scale bars: 100 μm, 50 μm, and 20 μm. All IF images show ASO-Cy3 (red), rod cell marker Rhodopsin (green), and nuclei stained with DAPI (blue). Data are presented as mean ± SD, with n ≥ 3. Statistical analysis was conducted by one-way ANOVA and Tukey’s multiple comparison test (D); *p < 0.05, ***p < 0.001, ****p < 0.0001.


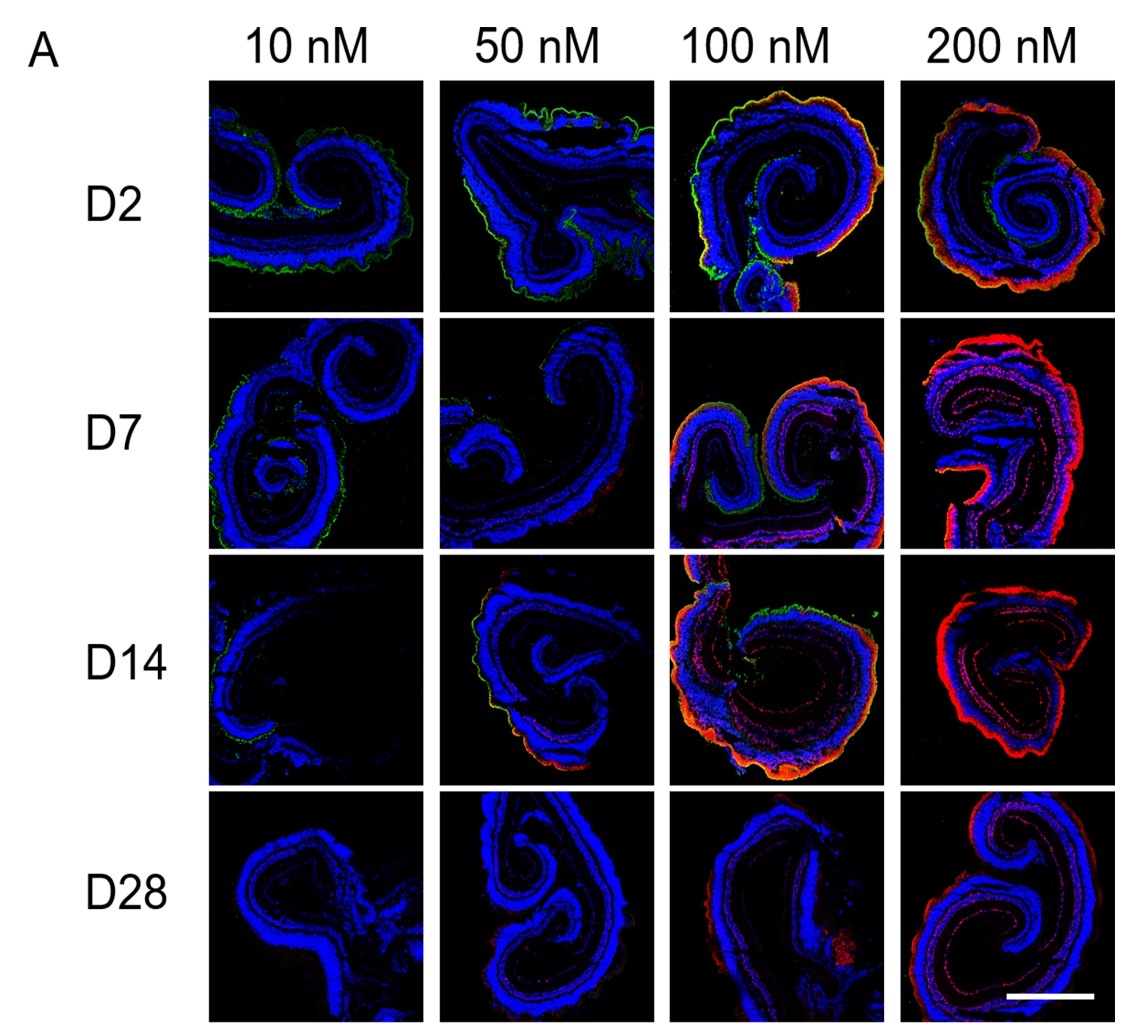


**Figure S2.** Fluorescence intensity distribution in retinal explants after ATR transfection with ASO-Cy3. (A) WT mouse neural retina explants cultured in suspension were treated with ATR-ASO-Cy3 reagent and transfected with varying concentrations (10, 50, 100, 200 nM) of ASO-Cy3. IF was performed on days 2, 7, 14, and 28 post-transfection, showing ASO-Cy3 (red), rod photoreceptor marker rhodopsin (green), and DAPI-stained nuclei (blue). Scale bar: 500 µm.


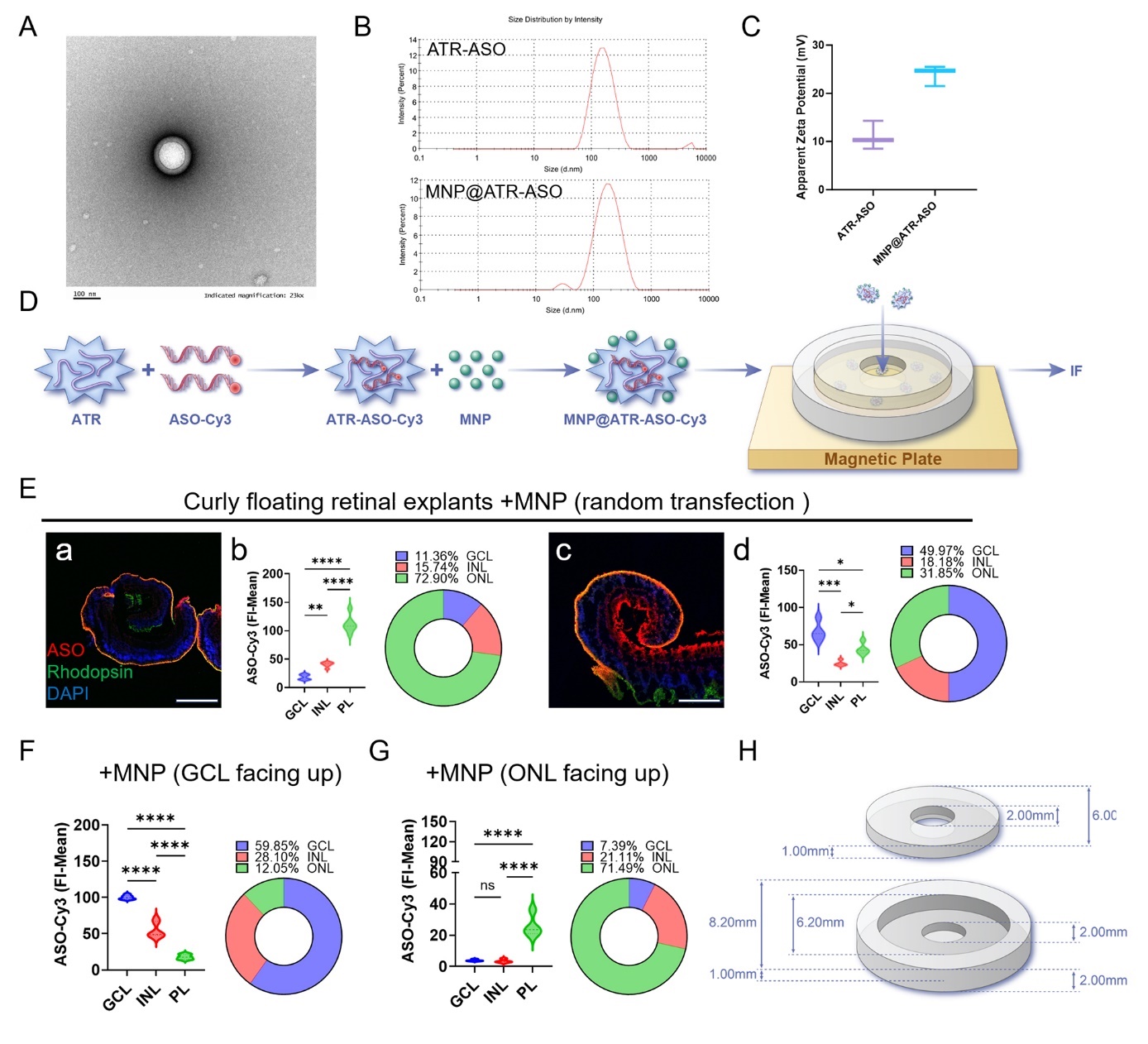


**Figure S3.** Characterization of the MNP@ATR-ASO Complex and Statistical Evaluation of ASO-Cy3 Delivery Efficiency and Targeting Across Various Retinal Layers in Murine Retinal Explants Facilitated by MNP. (A) The morphology of ATR-ASO was examined via transmission electron microscopy (TEM). (B) The mean hydrodynamic diameter of ATR-ASO and MNP@ATR-ASO particles was determined using dynamic light scattering (DLS), and (C) their surface electrostatic charge was assessed. (D) A schematic representation of the preparation process for the MNP@ATR-ASO-Cy3 complex is provided. (E) Statistical analysis of ASO-Cy3 fluorescence intensity (FI-Mean) was conducted for both the -MNP and +MNP groups in floating explant cultures. (F) Statistical evaluation of ASO-Cy3 FI-Mean distribution across different cellular layers with the GCL oriented upwards. (G) Statistical assessment of ASO-Cy3 FI-Mean distribution with the ONL oriented upwards. (H) The structural design of the collar and its alignment with the neuroretina, including the dimensions and nesting configuration of the upper and lower rings, is depicted. All bar graphs represent Mean±SD, with a sample size of n≥3. Statistical analyses were performed using univariate analysis of variance and the Tukey multiple comparison test (E-G). Significance levels are indicated as follows: *p < 0.05, ***p < 0.001, ****p < 0.0001.


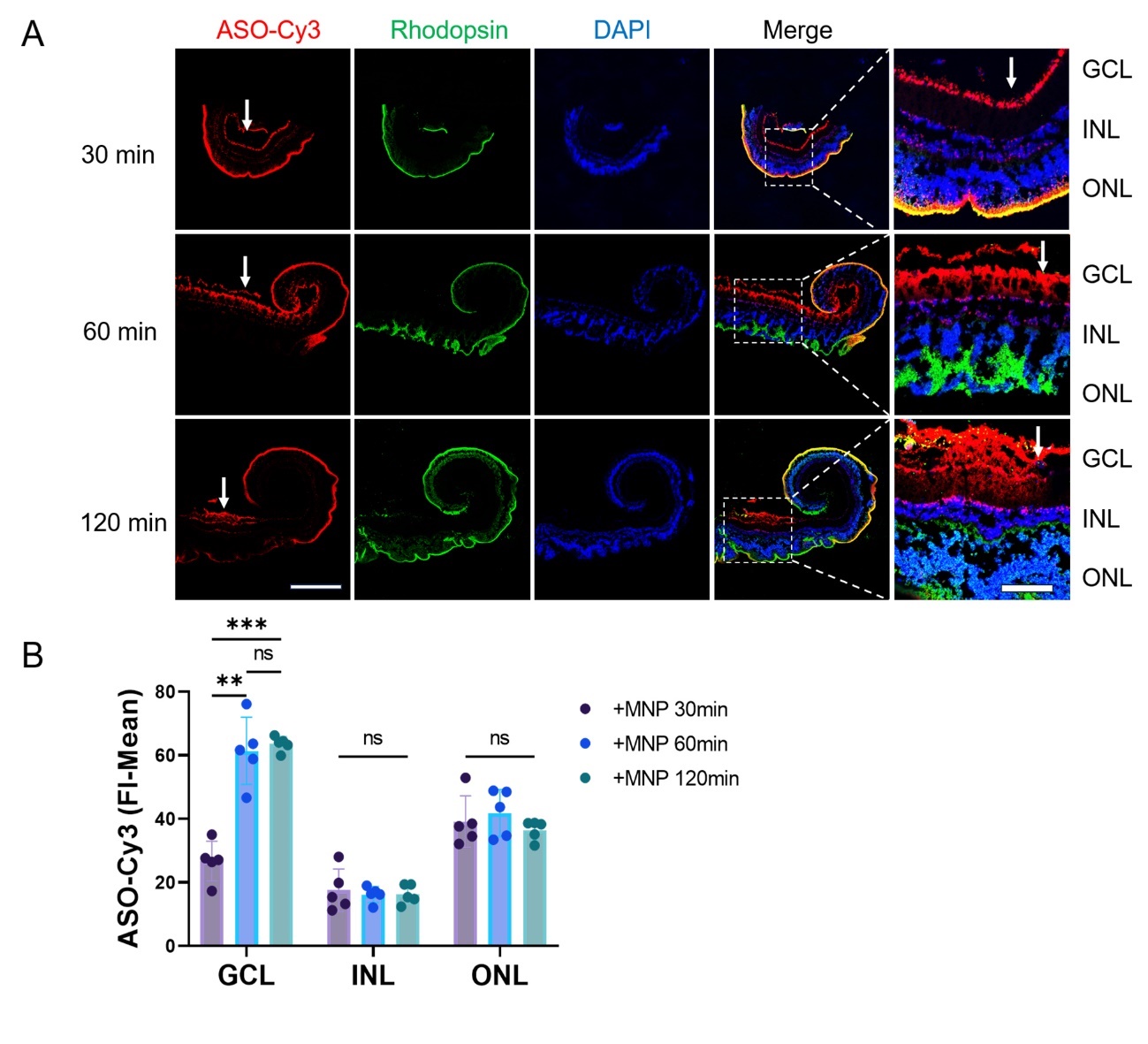


**Figure S4.** Fluorescence Distribution in Various Retinal Layers of Mouse Explants Transfected with MNP@ATR-ASO-Cy3 Complexes at Different Magnetic Exposure Times. (A) Immunofluorescence analysis of mouse retinal explants after 30, 60, and 120 minutes of magnetic exposure, showing ASO-Cy3 (red), rod cell marker rhodopsin (green), and nuclei stained with DAPI (blue). Images display ASO-Cy3 fluorescence distribution at different time points. Scale bars:500 µm (whole retina), 100 µm (magnified view). (B) Quantitative analysis reveals ASO-Cy3 FI-Mean across different cell layers at varying magnetization times. Data points represent individual values, and bars represent mean ± SD, n=5. Statistical analysis was conducted by two-way ANOVA with Tukey’s multiple comparison tests (B); ns p > 0.05, **p < 0.01, ***p < 0.001.


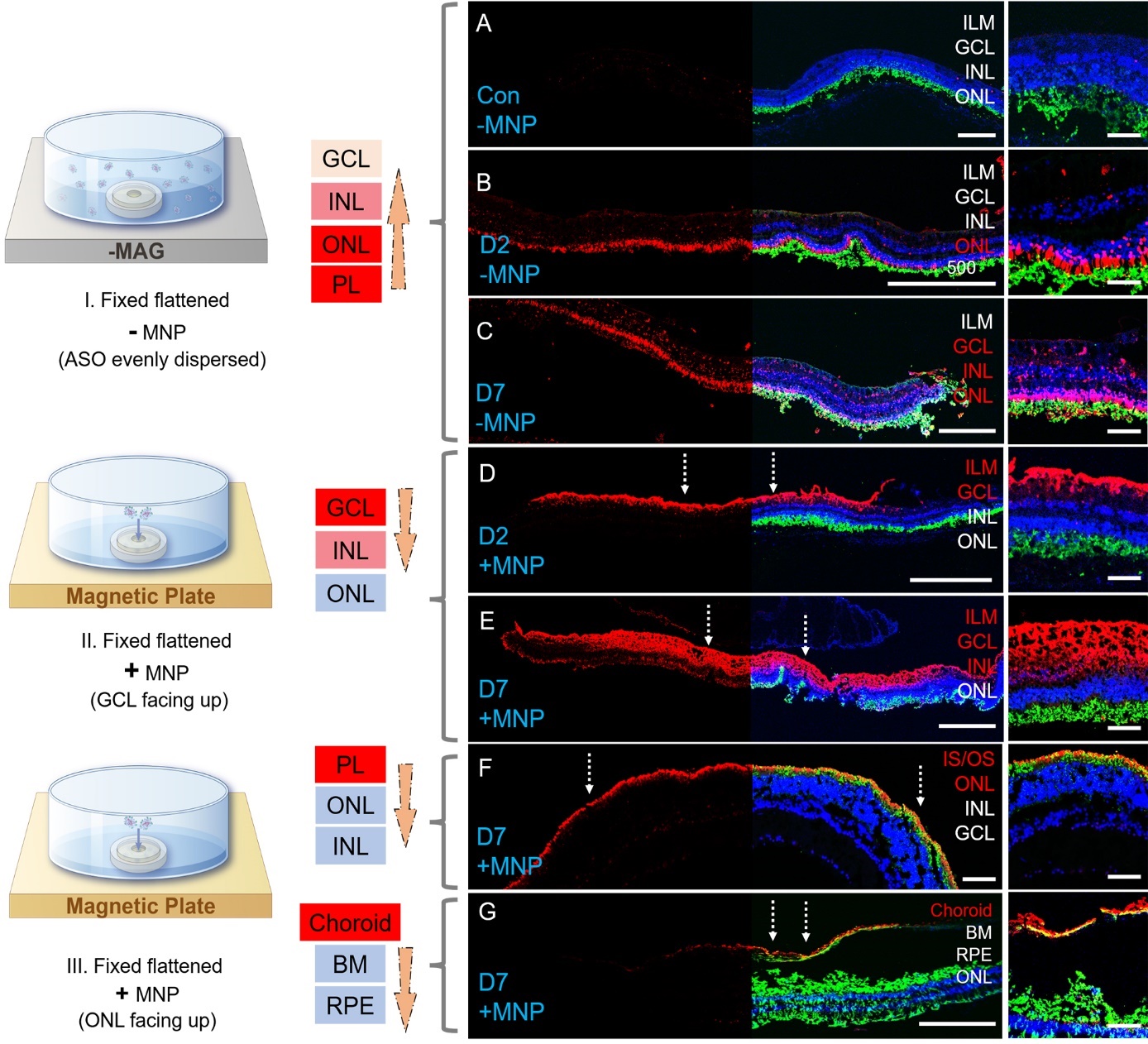


**Figure S5.** Overall Transfection Effects of MNP@ATR-ASO-Cy3 Complexes in Macaque Retinal Explants. Neural retinal explants from WT macaques were flattened and fixed. The experiment compared the distribution of ASO-Cy3 under different transfection strategies. (A) ATR transfection with non-fluorescent ASO complexes served as a control group. (B-C) ATR-ASO-Cy3 was evenly applied in medium, with IF analysis on day 2 (B) and day 7 (C) post-transfection. (D-E) In WT macaque retinal explants, with the GCL facing up, MNP@ATR-ASO-Cy3 was applied. IF images on day 2 (D) and day 7 (E). (F) Retinal explants were flattened with the ONL facing up, and MNP@ATR-ASO-Cy3 was applied. IF analysis on day 7 post-transfection. (G) Choroid-RPE-retina explants were fixed in wells with the choroid facing up, and MNP@ATR-ASO-Cy3 was applied. IF analysis on day 7 post-transfection. Scale bars: 500 µm (left), 100 µm (right).


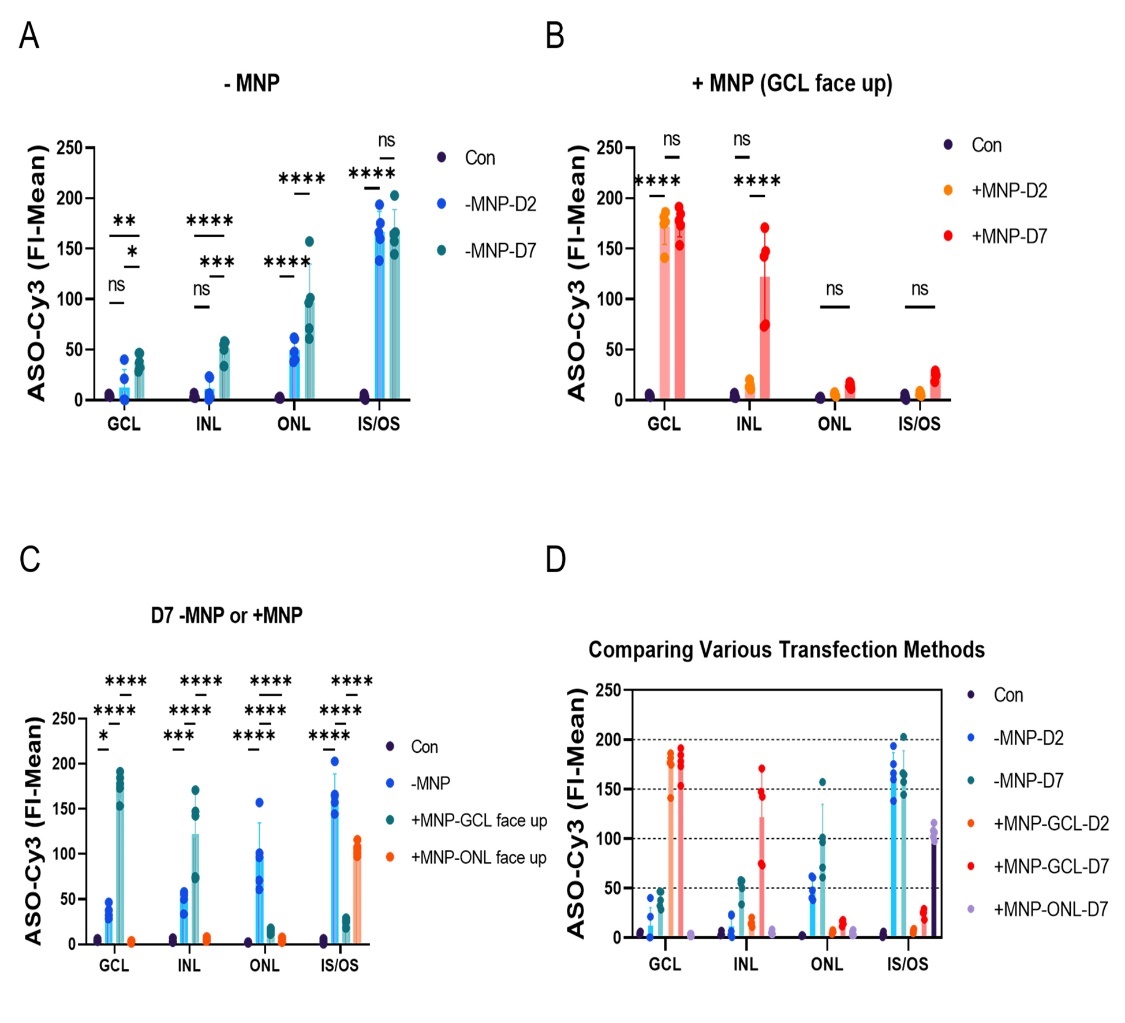


**Figure S6.** Comparison of Delivery Efficiency of Macaque Retinal Explants Using Various Transfection Methods. ATR transfection with non-fluorescent ASO complexes served as the control group. (A) ASO-Cy3 was delivered into macaque retinal explants using ATR transfection (-MNP), with fluorescence intensity distributions recorded on days 2 and 7 post-transfection. (B) When the GCL was oriented upwards, targeted transfection was performed with MNP. Fluorescence intensity distributions were recorded on days 2 and 7 post-transfection. (C) Three transfection methods were compared. Fluorescence intensity distributions were recorded on day 7 post-transfection. (D) The fluorescence intensity distributions were compared for ATR transfection alone on days 2 and 7, GCL facing up with MNP@ATR-ASO-Cy3 mixture on days 2 and 7, and ONL facing up using MNP@ATR-ASO-Cy3 mixture on day 7. Bars represent mean ± SD, n=5. Statistical analysis was conducted by two-way ANOVA and Tukey’s multiple comparison test (A-D); ns p > 0.05, *p < 0.05, **p < 0.01, ***p < 0.001, ****p < 0.0001.


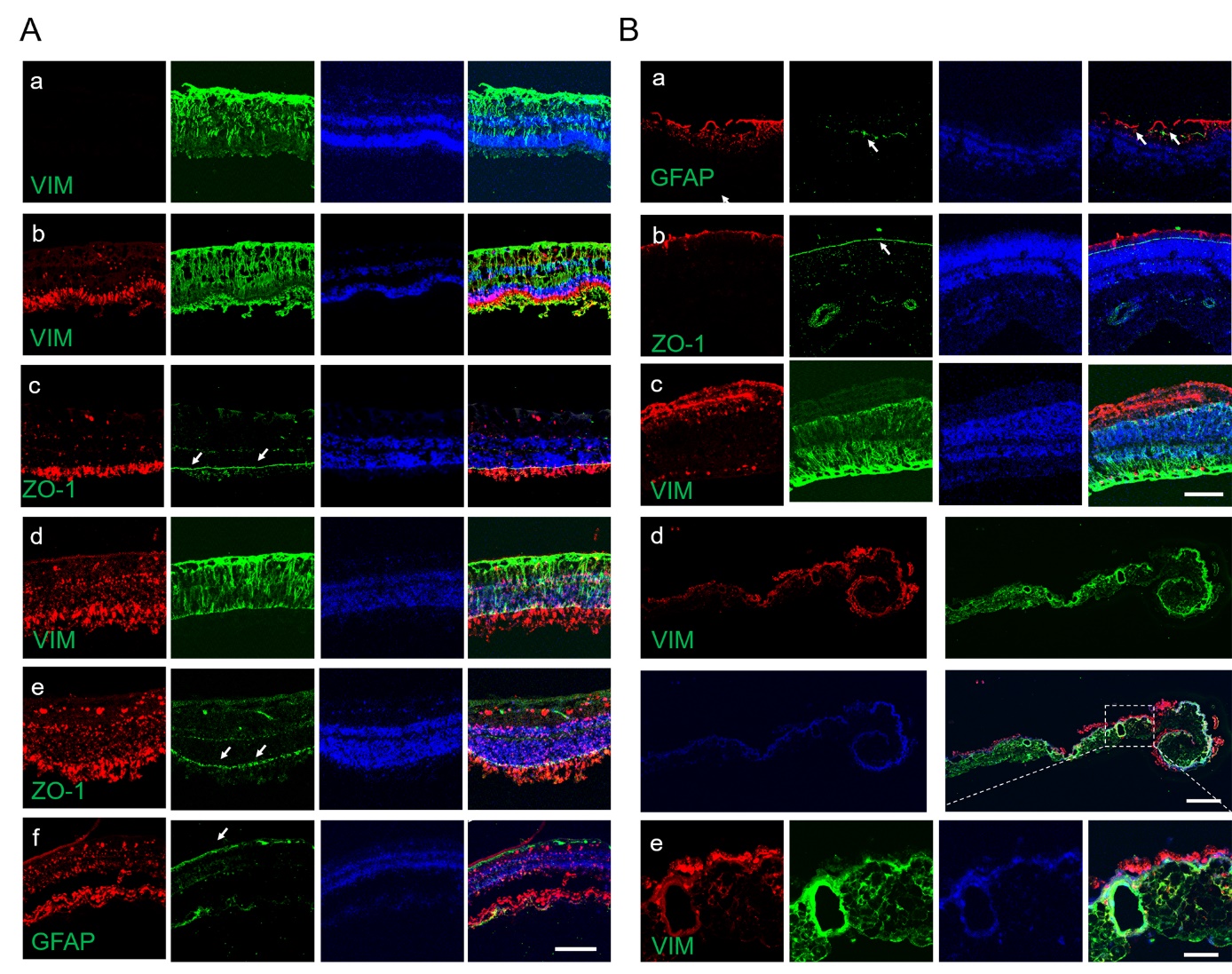


**Figure S7.** The transfection effects of MNP@ATR-ASO-Cy3 complexes on macaque retinal explants, assessed by IF for vimentin, GFAP, and ZO-1. (A-a) The explants without ASO transfection serve as a control group, with vimentin labeling Müller cells (green). (A-b, c) IF results on the second day after ATR transfection of ASO into the explants. (A-b) Vimentin staining shows that Müller cell fibers maintain their vertical structure from NFL to INL. (A-c) ZO-1 staining marks the position of the outer limiting membrane (OLM) (green). (A-d, e, f) IF results on the seventh day after ATR transfection of ASO in retinal explants. Scale bar: 200 µm. (B-a) For the GCL facing up group, IF images of GFAP staining (green) on day seven. (B-b, c) IF images of the ONL facing up group. Scale bar: 200 µm. (B-d, e) The choroid-RPE-retina complex is flattened and fixed, MNP-guided ASO-Cy3 successfully entering the choroid. Scale bar: 500 µm, 50 µm. In all IF images, the red signal represents ASO-Cy3, and cell nuclei are stained with DAPI (blue).


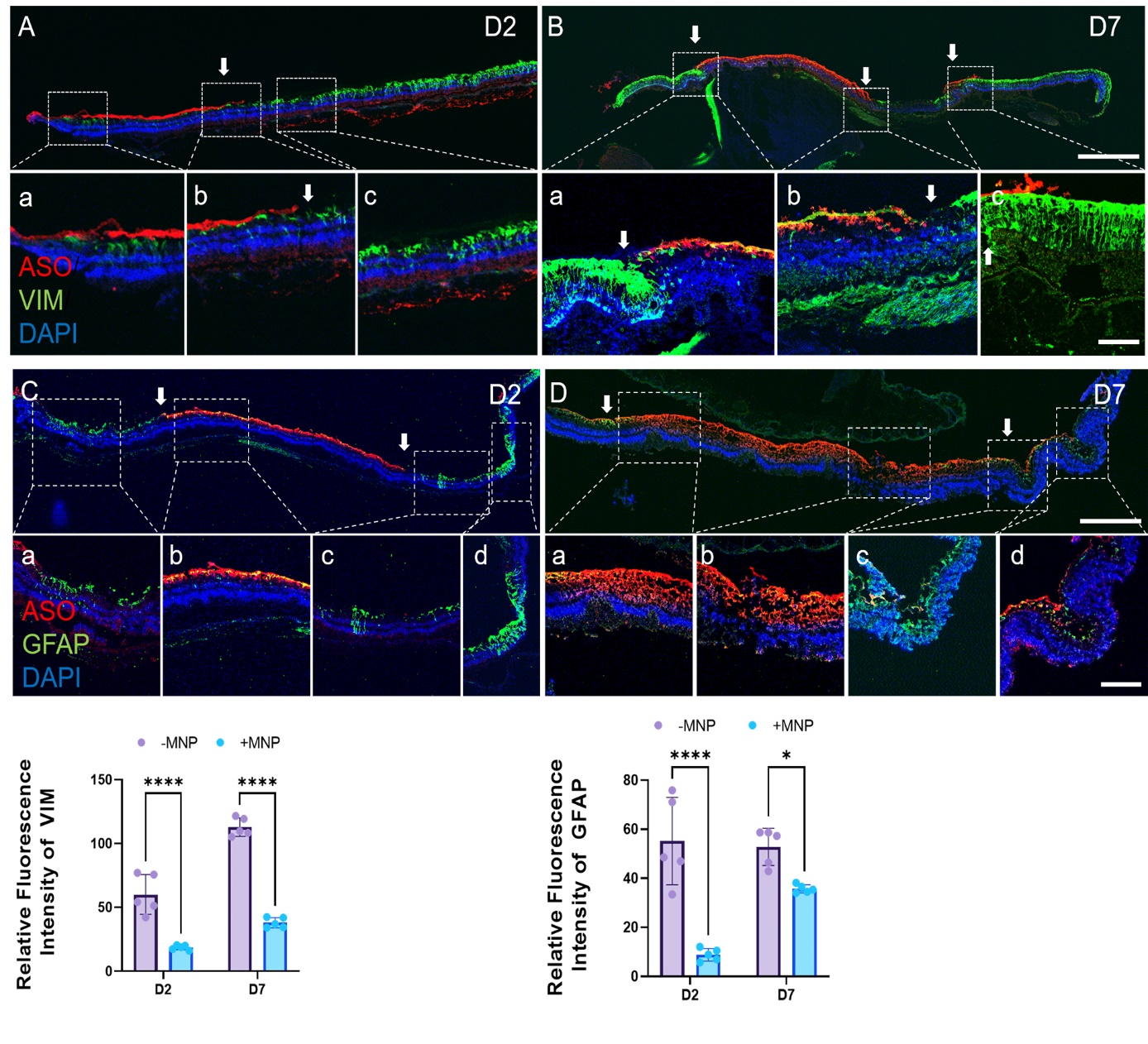


**Figure S8.** Immunofluorescence labeling of Müller cells in long fragments of macaque retinal explants using vimentin and GFAP. The GCL facing up with MNP@ATR-ASO-Cy3 complexes were applied. (A-B) IF staining with analyses conducted on days 2 (A) and 7 (B). (A-a, b, B-a, b) The white box indicates the area where ASO-Cy3 was successfully transfected show short, thick Müller cell fibers (weakened green signal). (A-c, B-c) In contrast, the non-transfected area displays longer Müller cell fibers (stronger green signal). Statistical analysis reveals significant differences between areas with +MNP and -MNP (n=5). (C-D) GFAP IF was performed to assess glial activation, with analyses conducted on days 2 (C) and 7 (D). (C-b, D-a, b) In regions where magnetic transfection was successful, glial activation is minimal. (C-a, c, d, D-c, d) Non-transfected areas show significant activation (enhanced green signal). Scale bars: 500 µm (original images) and 100 µm (magnified images). Bars represent mean ± SD. Statistical analysis was conducted by two-way ANOVA with Tukey’s multiple comparison tests; *p < 0.05, ****p < 0.0001.


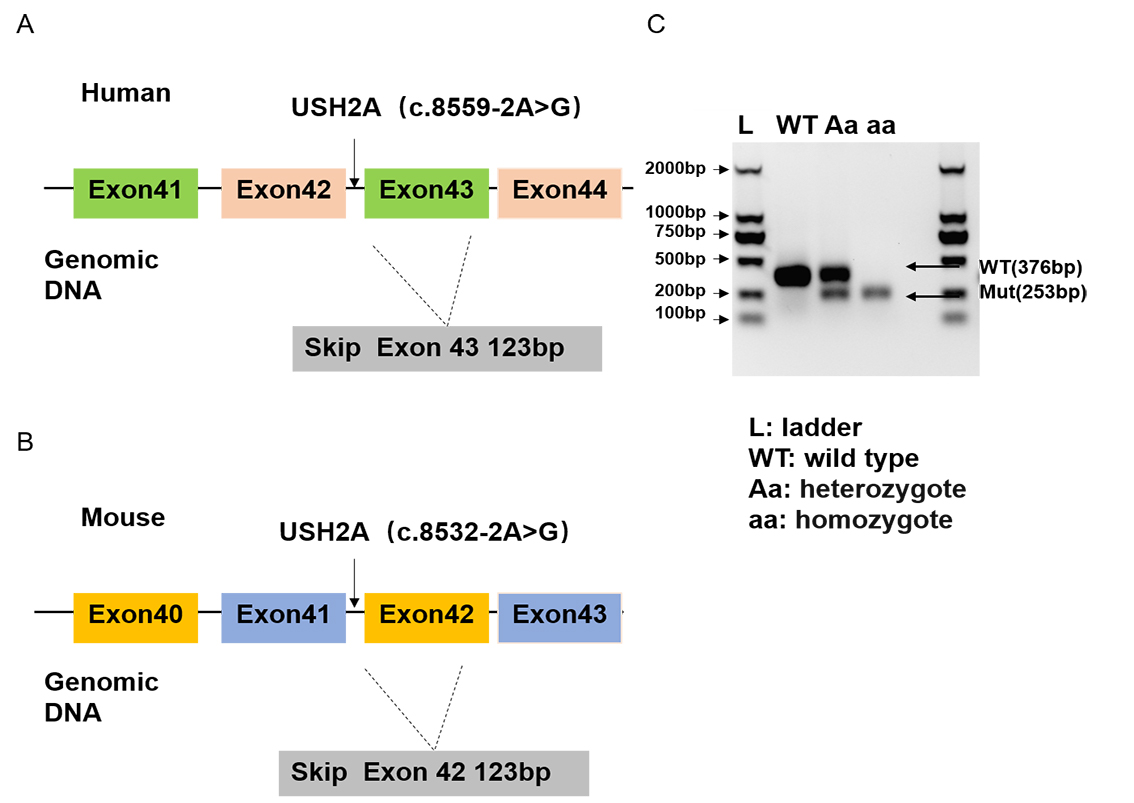


**Figure S9.** Validation of patient-specific splice abnormalities in humanized mouse retinal explants. (A) The diagram illustrates the splicing mechanism caused by the human USH2A mutation (c.8559-2A>G), which leads to the skipping of exon 43. (B) The diagram demonstrates the splicing mechanism caused by the humanized mouse USH2A mutation (c.8532-2A>G), which results in the skipping of exon 42. (C) RT-PCR results indicate splice variant abnormalities in retinal explants from mutant mice with the (c.8532-2A>G) mutation, leading to the skipping of exon 42.


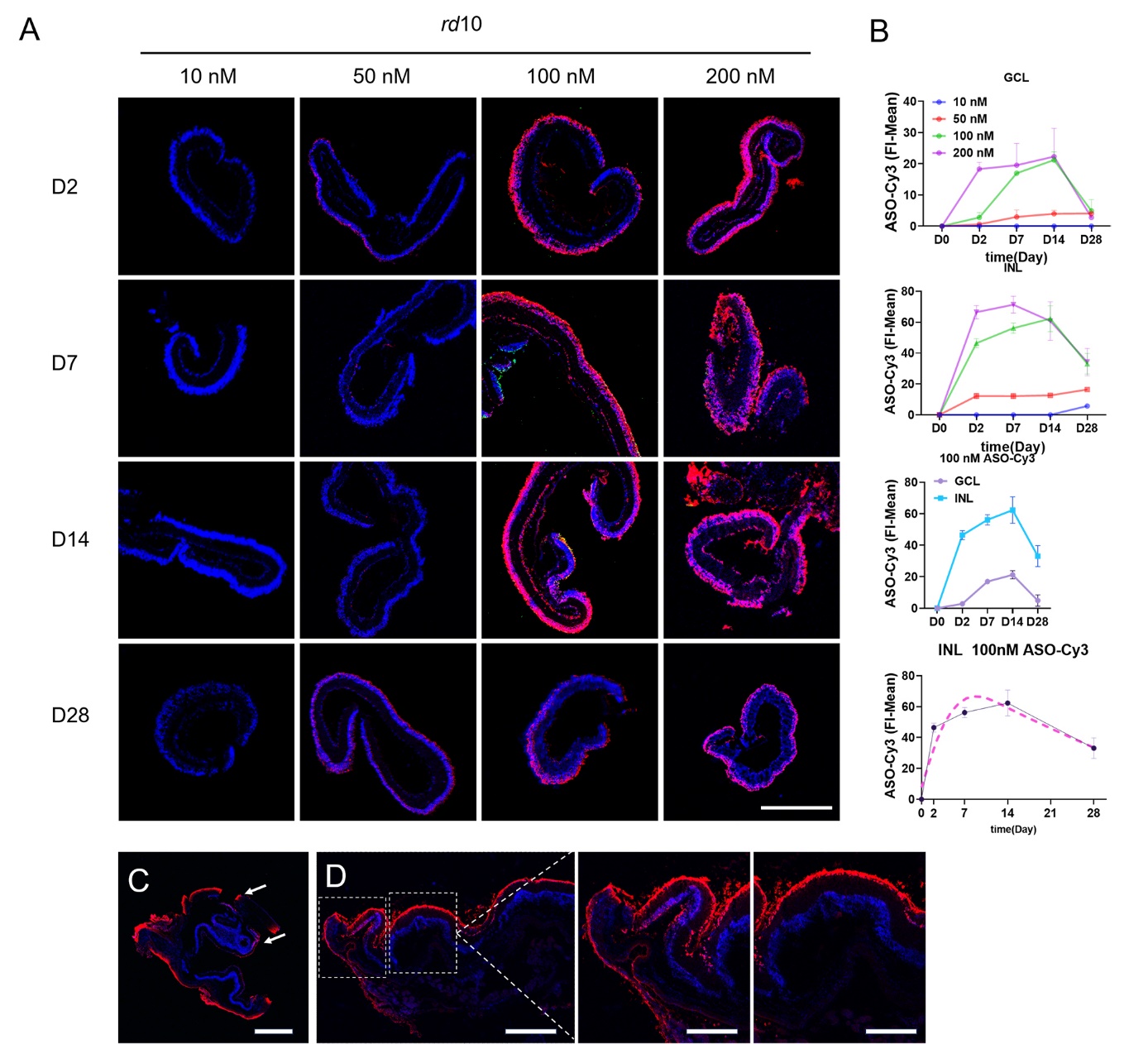


**Figure S10.** Transfection of ASO-Cy3 in vitro in rd10 mouse-derived retinal culture models. (A) Retinal explants isolated from rd10 mice were transfected with different concentrations (10, 50, 100, 200 nM) of ASO-Cy3 using ATR reagent. Immunostaining was performed on days 2, 7, 14, and 28 post-transfection. Scale bar: 500 µm. (B) Transfection efficiency of ASO-Cy3 was analyzed by comparing fluorescence intensity at different concentrations and time. A curve fitting of FI-Mean over time in the INL was performed. (C-D) Organotypic neuroretina-RPE-choroid explants from rd10 mice were transfected with ASO-Cy3. ASO-Cy3 (red), rod cell marker rhodopsin (green), and nuclei stained with DAPI (blue). Scale bar: 500 µm.


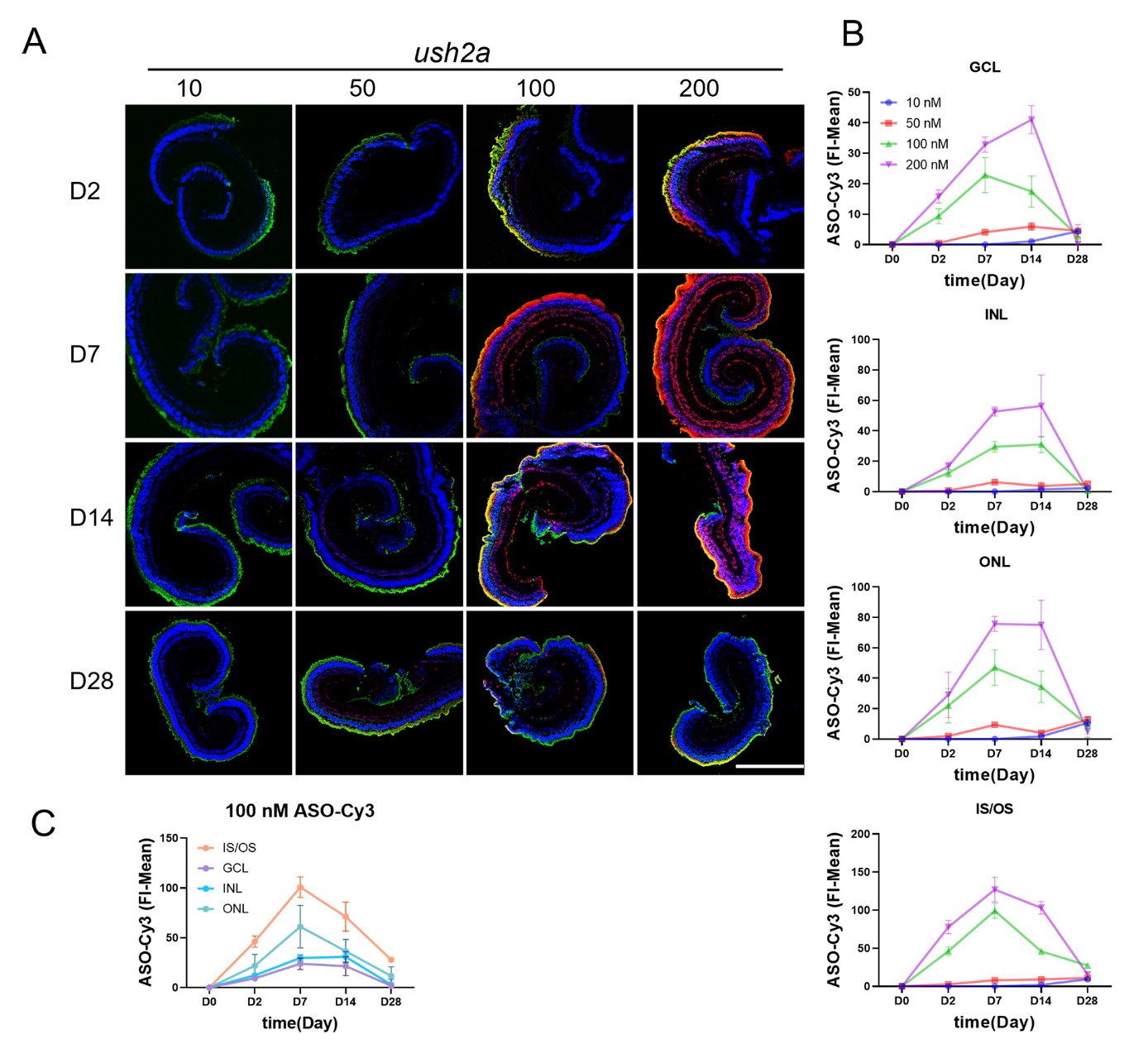


**Figure S11.** Changes in Fluorescence Intensity of ASO-Cy3 at Different Concentrations and Transfection Times in USH2A Mouse Retinal Explants. (A) Retinal explants from USH2A mice were transfected with different concentrations (10, 50, 100, 200 nM) of ASO-Cy3 using ATR. Immunostaining analysis was performed on days 2, 7, 14, and 28. ASO-Cy3 (red), rod cell marker rhodopsin (green), and nuclei stained with DAPI (blue). Scale bar: 500 µm. (B) The FI-Mean of each retinal cell layer at different time points was compared. Curve fitting was performed on the FI-Mean in the IS/OS layer. Bars represent Mean ± SD, n≥3.


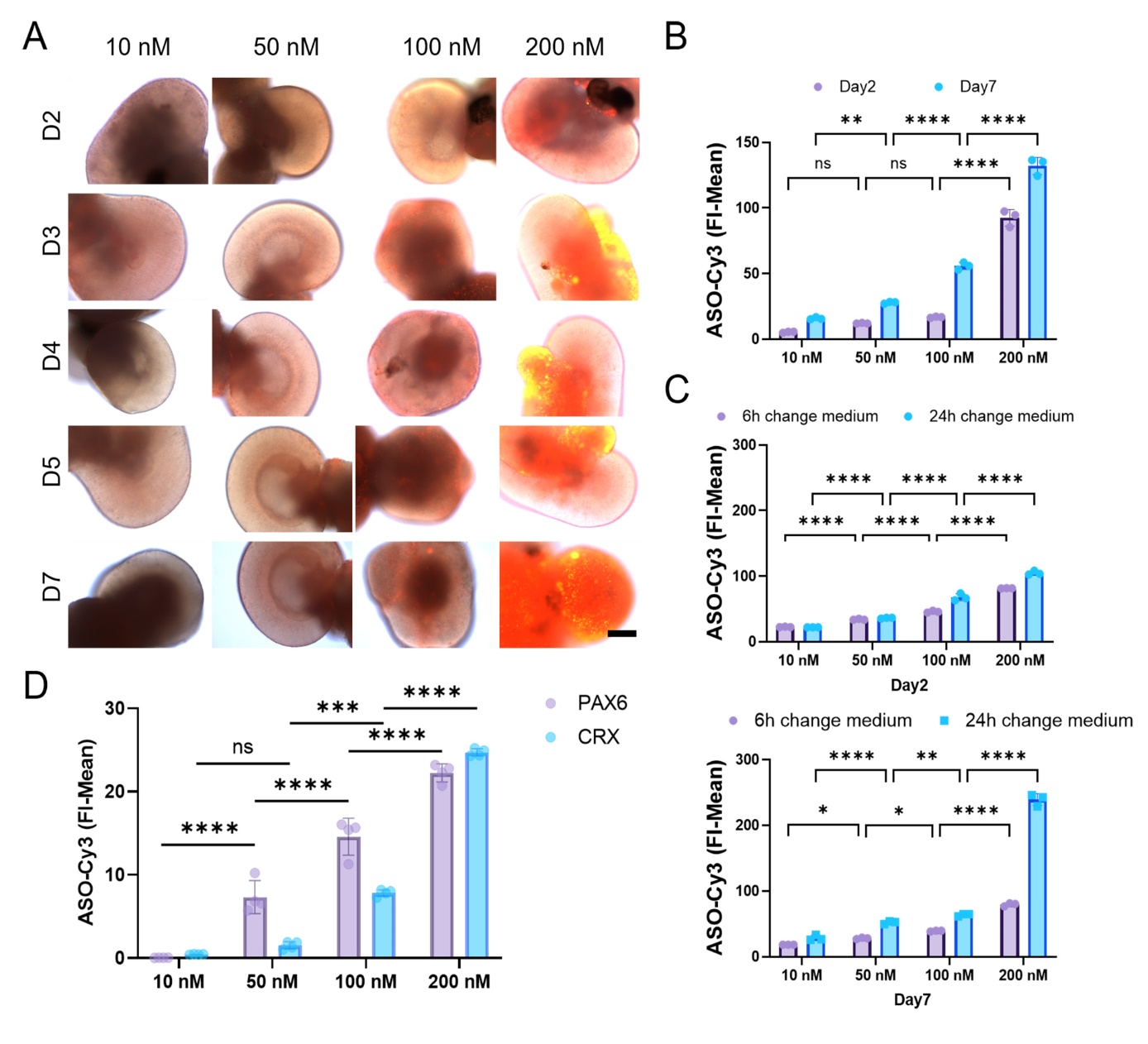


**Figure S12.** Transfection efficiency of ASO using ATR with gradient concentrations into hROs. (A) Fluorescence imaging results of hROs treated with ASO-Cy3 at different concentrations on days 2, 3, 4, 5, and 7. Scale bar: 250 µm. (B) The statistical graph shows the FI-Mean of ASO-Cy3 on days 2 and 7 post-transfection. n=3. (C) The statistical graph shows the FI-Mean changes of ASO-Cy3 at different transfection gradient concentrations, comparing the replacement of fresh medium at 6 hours or 24 hours post-transfection on days 2 and 7. n=3. (D) The statistical graph represents the quantitative fluorescence analysis on day 7 post-transfection, showing the FI-Mean of ASO-Cy3 in the PAX6 and CRX groups. n=4. Bars are presented as mean ± SD. Statistical analysis was conducted by two-way ANOVA with Tukey’s multiple comparison tests (B-D); ns p > 0.05, *p < 0.05, **p < 0.01, ***p < 0.001, ****p < 0.0001.


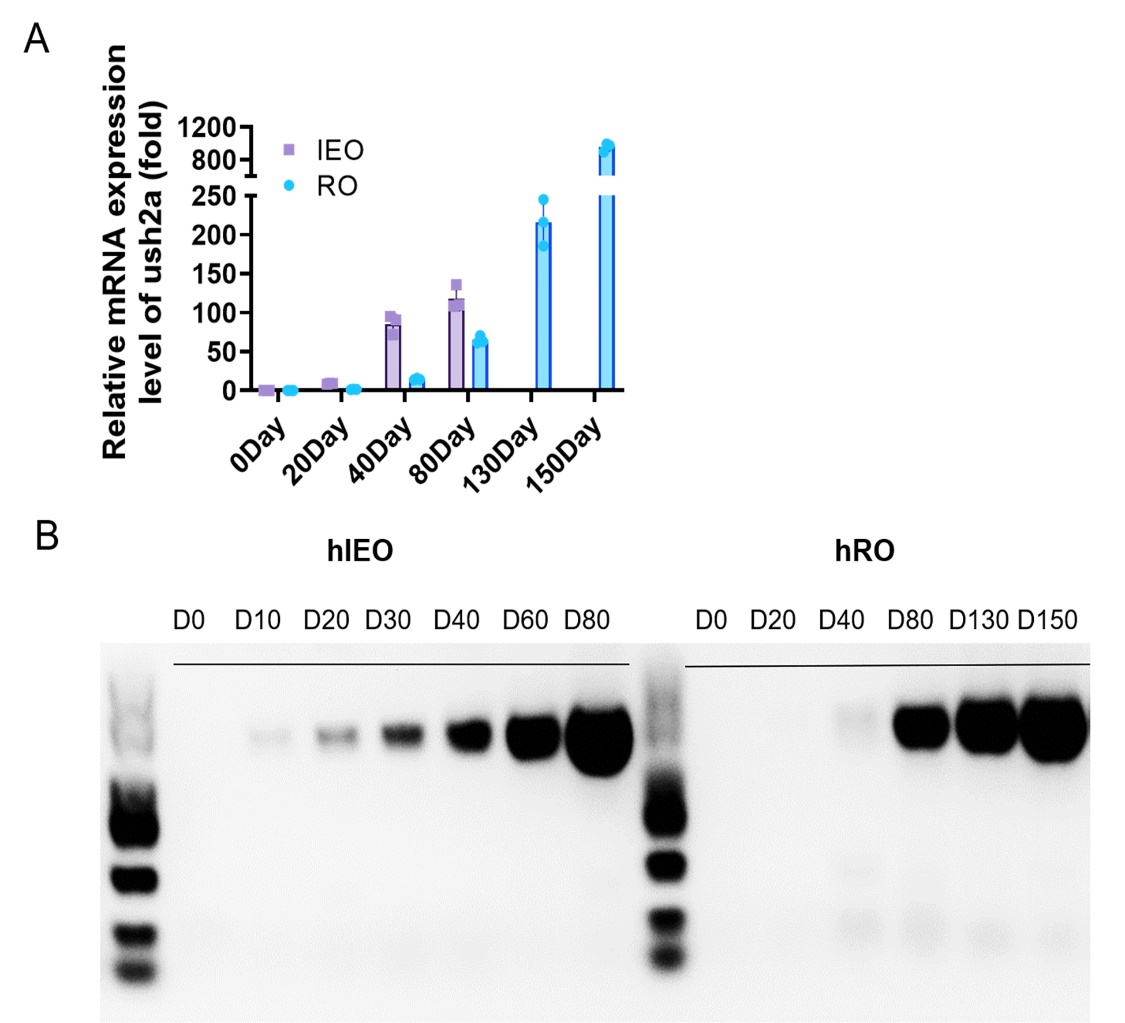


**Figure S13.** Comparative Analysis of USH2A Transcript Expression Levels in hIEOs and hROs at Different Differentiation Stages. (A) qPCR analysis of transcript expression levels in hIEOs and hROs at different stages of differentiation. (Mean ± SD, n ≥ 5 independent cultures). (B) Qualitative analysis of human USH2A exon 13 expression in hIEOs and hROs at different stages of differentiation using RT-PCR. Gel electrophoresis results show clear bands in hIEOs at day 30, while hROs show clear bands only at 60 to 80 days, (n ≥ 5 independent cultures).


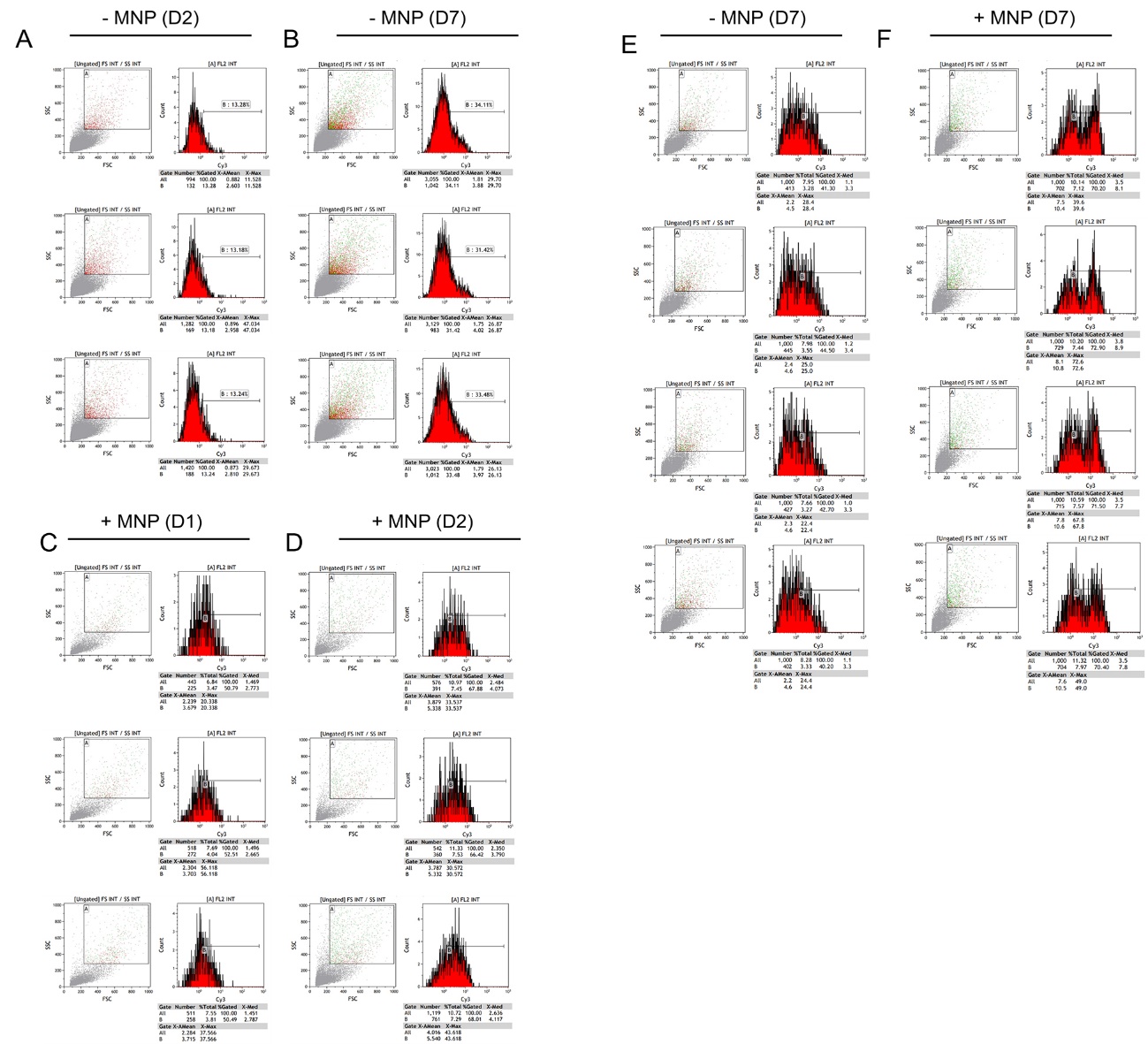


**Figure S14.** Comparative flow cytometry analysis of hROs and hIEOs post-transfection between -MNP and +MNP groups. (A-D) The hROs are categorized into four groups: -MNP Day 2, -MNP Day 7, +MNP Day 1, and +MNP Day 2. (E, F) The hIEOs are divided into two groups: -MNP Day 7 and +MNP Day 7. Results include maximum fluorescence intensity (X-Max), median fluorescence intensity (X-Med), and percentage of positive cells (%Gated).

# Supplementary Table

**Table S1.** Transfection reagents used in this study

| Transfection Reagent | Abbreviations | Item No. | Manufacturers | Volume (µl)^a^ |
| --- | --- | --- | --- | --- |
| Advanced DNA RNA Transfection Reagent | ATR | AD600025 | ZETA LIFE, USA | 5 |
| RNATransMate | RTM | E607402-0100 | BBI, China | 10 |
| Lipofectamine™ RNAiMAX | LRM | 13778100 | Invitrogen™, USA | 30 |
| siTran siRNA Transfection | SIT | TT320001 | OriGene, Rockville, MD, USA | 10 |
| INTERFERin | INT | 101000036 | Polyplus, France | 20 |

^a)^Volume of transfection reagent used for 100 nM ASO (µl)

**Table S2.** ASO sequences used in the study

| Name | System | Sequence | Remark |
| --- | --- | --- | --- |
| QR-421a | human | 5’-AGCUUCGGAGAAAUUUAAAUC  -3’ | MOE^a^ |
| mQR-421a | mouse | 5’-AACUCUGGAGGAAUUUAAAUC  -3’ | MOE^a^ |
| Control | human/mouse | 5’-AUAGUAACGGAUUGAGG-3’ | MOE^a^ |

^a)^MOE: fully phosphorothioated backbone and 2’ O-methoxyethyl RNA bases

**Table S3.** Neural retinal Explant Medium (NRM)

| Reagent | Proportion | Item No. | Manufacturers |
| --- | --- | --- | --- |
| Neurobasal A |  | 21103049 | Gibco |
| B-27 | 2% | 17504044 | Gibco |
| N2 | 1% | 17502048 | Gibco |
| P/S（10000 U/ml） | 1% | 15140-122 | Gibco |
| GlutaMax | 0.4% | 35050061 | Gibco |

**Table S4.** hROs differentiation protocol

| Day | Reagent | Proportion | Manufacturers |
| --- | --- | --- | --- |
| Day -2 | hiPSC | mTeSR1 | Stemcell Technologies |
|  |  | Matrigel | BD Biosciences |
| Day 0 | Dissociated into small clumps | mTeSR1 | Stemcell Technologies |
|  |  | 10 µM Blebbistatin | Sigma |
| Day 1 | mTeSR1/NIM | 3:1 | |
| Day 2 |  | 1:1 | |
| Day 3 |  | 1:3 | |
| Day 4-7 | NIM | | |
| Day 7 | Seeded onto Matrigel coated dishes | Matrigel | BD Biosciences |
| Day 16 | DMEM/F12(3:1) | | |
|  | B27 (without vitamin A,), | 2% | Invitrogen |
|  | NEAA | 1x | Gibco |
|  | antibiotic-antimycotic | 1% | Gibco |
|  | the medium was changed daily | | |
| Formation of 3-D Retinal Cups | | | |
| 4th week | Horseshoe-shaped neural retina domains were manually detached | | |
|  | DMEM/F12 (3:1) |  | Gibco |
|  | B27, | 2% | Invitrogen |
|  | NEAA | 1x | Gibco |
|  | Antibiotic-antimycotic | 1% | Gibco |
| Formed 3-dimesional retinal cups. The medium was changed twice a week. | | | |
| Day 42 | Fetal bovine serum | 10% | Gibco |
|  | Taurine | 100 µM | BD Biosciences |
|  | Glutamax | 2 mM | Gibco |

**Table S5.** Neural induction medium (NIM)

| Dulbecco’s modified eagle medium  (DMEM)/F12 (1:1) | Gibco |  |
| --- | --- | --- |
| N2 supplement | Invitrogen | 1% |
| minimum essential media-non  essential amino acids (NEAA) | Gibco | 1x |
| heparin | Sigma | 2 µg/ml |
| Normocin | Invivogen | 100 µg/ml |

**Table S6.** hIEOs differentiation protocol

| Day | Reagent |  | |
| --- | --- | --- | --- |
| Day -2-0 | iPSCs | E8 Medium,V-bottom | |
| Day 0 | CDM Medium | FGF-2 | 4 ng/ml |
|  |  | SB-431542 | 10 µM |
|  |  | BMP 4 | 2.5 ng/ml |
|  |  | GFR Matrigel | 2% |
| Day 4 |  | U-bottom |  |
|  |  | FGF-2 | 50 ng/ml |
|  |  | LDN-193189 | 200 n M |
| Day 8 |  | CHIR99021 | 3 µM |
| Day 12 | OMM Medium | CHIR99021 | 3 µM |
|  |  | GFR Matrigel | 1% |
| Day18 | OMM Medium | CHIR99021 | 3 µM |
| Day20 | OMM Medium | 65 rpm shake |  |

**Table S7.** Chemically-Defined Differentiation Medium (CDM)

| Component | Supplier | Cat. No. | Stock Concentration | Final Concentration | Volume used |
| --- | --- | --- | --- | --- | --- |
| Ham's F12  GlutaMax | Gibco | 31765-035 | - | 49%(v/v) | 100 ml |
| IMDM GlutaMax | Gibco | 31980-030 | - | 49%(v/v) | 100 ml |
| Chemically-Defined Lipid | Gibco | 11905-031 | 100x | 1X | 2 ml |
| BSA | Sigma | A1470 | - | 5 mg/ml | 1 g |
| Insulin | Sigma | 19278 | 10 mg/ml | 7 µg/ml | 140 μl |
| Transferrin | Sigma | T8158 | 20 mg/ml | 15 µg/ml | 150 μl |
| 1-thioglycerol | Sigma | M6145 | 11.5 M | 450 µM | 8 μl |
| Normocin | Invivogen | Ant-nr-1 | 50 mg/ml | 100 µg/ml | 400 μl |

**Table S8.** Organoid Maturation Medium (OMM)

| Component | Supplier | Cat. No. | Stock  Concentration | Final  Concentration | Volume (50 ml) |
| --- | --- | --- | --- | --- | --- |
| Adv DMEM/F12 | Gibco | 12491-015 | - | 49% (v/v) | 24.5 ml |
| Neurobasal | Gibco | 21103-049 | - | 49% (v/v) | 24.5 ml |
| N2 supplement | Gibco | 17502-048100x | 100x | 0.5x | 250 μl |
| B27 -Vitamin A | Gibco | 12587-01050x | 50x | 0.5x | 500 μl |
| GlutaMAX | Gibco | 35050-079100x | 100x | 1x | 500 μl |
| Mercaptoethanol | Gibco | 21985-015 | 55 mM | 0.1 mM | 91 μl |
| Normocin | Invivogen | Ant-nr-1 | 50 mg/ml | 100 µg/ml | 100 μl |

**Table S9.** Primers for qPCR

| Gene | Forward Primer （5’-3’） | Reverse Primer （5’-3’） |
| --- | --- | --- |
| USH2A | CAAACAGCAGTGCTTGGGTA | TGCGGAAGTCACATTGGTTA |
| β-actin | CTCCATCCTGGCCTCGCTGT | GCTGTCACCTTCACCGTTCC |
| BAX | CACAGGTTGGCATTAGGAAGGC | GGTGAGCGAGGCGGTGAG |
| BCL-2 | CTGTGCTGCTATCCTGCCAAG | GTCTGTGTTCTTCATCGTTACTTCAAG |
| GFAP | GGCAGGATGGAGCGGAGAC | AGGAGAAGCGTGGCATGGTAC |
| HSP70 | CATGGTGCTGACGAAGATGAAGG | GCCTGCCGCTGAGAGTCG |
| GAPDH | ATCACTGCCACCCAGAAGAC | ACCAGGAAATGAGCTTGACA |

**Table S10.** Primers for RT-PCR

| Regions | Primer names | Primer sequence |
| --- | --- | --- |
| mouse USH2A gene  Exons 11-13 | *Ush2a* exon 11-13 mouse  full length: 900 bp | 5’-AGTGCCTTCAGTGCCAGGAT-3’ |
|  | Exon12：642 bp | 5’-AAGAGCACTGTCCCGTGACA-3’ |

**Table S11.** Primary and Secondary Antibodies Used for Immunohistochemical Analysis

| Antigen | Species | Dilution | Source |
| --- | --- | --- | --- |
| Vimentin | Mouse | 1:500 | santa cruz biotechnology |
| NeuN | Rabbit | 1:200 | Novus |
| GFAP | Rabbit | 1:1000 | Novus |
| Rhodopsin | Mouse; | 1:100 | santa cruz biotechnology |
| PAX6 | Mouse | 1:500 | santa cruz biotechnology |
| CRX | Mouse | 1:100 | santa cruz biotechnology |
| Sox2 | Mouse | 1:100 | BD Biosciences |
| PAX8 | Rabbit | 1:100 | Abcam |
| Myo7a | Mouse | 1:100 | santa cruz biotechnology |
| Anti-mouse-488 | Goat | 1:200 | Invitrogen |
| Anti-rabbit-488 | Goat | 1:200 | Invitrogen |
